# Feed Intake of Growing Dairy Heifers Raised under Tropical Conditions: A Model Evaluation Using Meta-Analysis

**DOI:** 10.3390/ani11113181

**Published:** 2021-11-07

**Authors:** Marcos Busanello, Debora Gomes de Sousa, Filipe Araújo Canedo Mendonça, Veridiana Lourenço Daley, Rodrigo de Almeida, Carla Maris Machado Bittar, Dante Pazzanese Duarte Lanna

**Affiliations:** 1 Department of Animal Science, College of Agriculture “Luiz de Queiroz”/University of São Paulo—ESALQ/USP, Piracicaba 13418-900, SP, Brazil; deborasousa@usp.br (D.G.d.S.); fmendonca@usp.br (F.A.C.M.); carlabittar@usp.br (C.M.M.B.); dplanna@usp.br (D.P.D.L.); 2 Department of Dairy Science, Virginia Tech, Blacksburg, VA 24061, USA; veridianalsouza@gmail.com; 3 Department of Animal Science, Federal University of Paraná, Curitiba 80035-050, PR, Brazil; ralmeida@ufpr.br

**Keywords:** dry matter intake, dairy heifers, *Bos taurus*, *Bos indicus*, empirical models

## Abstract

**Simple Summary:**

Our study evaluated seven DMI models for dairy heifers grouped by their genotypes (*Bos taurus* or crossbred *Bos taurus* × *Bos indicus*) raised under tropical climatic conditions. The HHJ and OFNLin DMI models performed better for *Bos taurus* heifers, whereas the STA model performed better for crossbred heifers. NRC, HH, QUI, and OFLin DMI models had significant significant slope bias, mean bias, or both.

**Abstract:**

Several models for predicting dry matter intake (DMI) of replacement dairy heifers have been developed; however, only a few have been evaluated using data from heifers of different breeds raised under tropical conditions. Thus, the objective of this study was to evaluate the DMI equations for dairy heifers managed under tropical conditions. A total of 230 treatment means from 61 studies using dairy heifers (*n* = 1513 heifers, average body weight = 246 kg) were used. The animals were grouped into two groups based on their genetics: (1) *Bos taurus* (Holstein, Jersey, Brown Swiss, and Holstein × Jersey) and (2) crossbred (*Bos taurus* × *Bos indicus*). Seven previously published DMI equations (HH, HHJ, QUI, STA, 2001 NRC, OFLin, and OFNLin) for heifers were evaluated using mean bias, slope bias, mean squared prediction errors (MSPE) and its decomposition, and other model evaluation statistics. For *Bos taurus* heifers, our results indicated that OFNLin and HHJ had lower mean bias (0.13 and 0.16 kg/d, respectively) than other models. There was no significant slope or mean bias for HHJ and OFNLin (*p* > 0.05), indicating agreement between the observed and predicted DMI values. All other models had a significant mean bias (*p* < 0.05), whereas the QUI model also presented a significant slope bias (*p* < 0.02). For crossbred heifers, the STA equation was the only one that did not present mean and slope bias significance (*p* > 0.05). All other DMI models had significant mean bias when evaluated using crossbred data (*p* < 0.04), and QUI, OFLin, and OFNLin also presented significant slope bias (*p* < 0.01). Based on our results, predictions from OFNLin and HHJ best represented the observed DMI of *Bos taurus* heifers (MSPE ≤ 1.25 kg^2^/d^2^, mean bias ≤ 0.16 kg/d), whereas STA was the best model for crossbred heifers (MSPE = 1.25 kg^2^/d^2^, mean bias = 0.09 kg/d). These findings indicate that not all available models are adequate for estimating the DMI of dairy heifers managed under a tropical climate, with HHJ and OFNLin for *Bos taurus* and STA for crossbreds being the most suitable models for DMI prediction. There is evidence that models from *Bos taurus* heifers could be used to estimate the DMI of heifers under tropical conditions. For heifer ration formulation is necessary to consider that DMI is influenced by breed, diet, management, and climate. Future work should also include animal genetic and environmental variables for the prediction of DMI in dairy heifers.

## 1. Introduction

Dry matter intake (DMI) is one of the most important animal health and performance indicators of dairy cattle [1]. In the last 30 years, animal genetics and nutritional requirements have changed, resulting in increased feed efficiency. Over the past decade, many studies have reported mechanisms that can affect DMI in ruminants, mainly for lactating dairy cows. Although substantial advances have been made in this area, it is necessary to consider variation in the actual DMI within and between animals, which models and science still cannot explain.

An important aspect of ration formulation is that DMI can be considered input (if measured at the farm) or output (if estimated by models). Dairy nutritionists usually use models to estimate the DMI of heifers because many dairy farmers do not have a feed measurement system because of the high implementation cost. Thus, several intake models have been developed and used in feed formulations for dairy cattle, in which animal characteristics, dietary components, environmental conditions, and management factors are frequently used as inputs.

For dairy heifers, DMI models have been used to calculate the nutrients supplied from the diet or to predict the nutrient requirements for maintenance, growth, and pregnancy, and to create growth curves for different breeds. Many factors may affect the DMI of heifers; for example, the number of pregnancy days is negatively correlated with feed intake around calving time [2,3]. High environmental temperatures can also decrease DMI [4]. Although environmental temperature and humidity can affect DMI, only a few models for dairy heifers include environmental variables as inputs because limited data are available [5].

Since 1980, DMI models for heifers have been developed using different modeling approaches. First, DMI models for heifers were described by Stallings et al. (1985) [6] and Quigley et al. (1986a) [7]. Stallings et al. (1985) [6] used body weight (BW) as an independent variable to fit a quadratic polynomial regression, but this equation was not adopted by the Dairy National Research Council (NRC) committee (1989, 2001) [8,9] because of its significant bias. At that time, two DMI models developed by Quigley et al. (1986a) [7] and the Beef NRC committee (1996) [10] were considered adequate. Although the model developed by Quigley et al. (1986a) [7] had low bias when evaluated for dairy heifers, the Dairy NRC (2001) [9] adopted the Beef NRC (1996) [10] equation for dairy heifers because it was evaluated using a larger dataset.

Overall, models for estimating the DMI of dairy heifers have been developed and evaluated using Holstein or beef cattle data exclusively. Moreover, most equations were fitted using linear regression, with or without quadratic or cubic terms. Nonlinear models have also been used to predict DMI in dairy heifers, such as those proposed by Hoffman et al. (2008) [11] and Oliveira and Ferreira (2016) [5]. In recent years, advances in computational techniques have allowed fitting nonlinear mixed-effects models to biological data to better understand nonlinear relationships between variables using large amounts of data.

In terms of animal genetics, nutrition models developed for Holstein cows are often adopted for crossbred cows worldwide because of the limitation of intake and nutritional requirement equations for different breeds (Brown Swiss, Jersey, Gyr, other zebu and crossbred cattle, etc.). Souza (2015) [12] developed an equation to predict DMI of lactating crossbred cows using metabolic body weight (BW^0.75^), with 4% fat-corrected milk, and the week of lactation as independent variables. That study reported differences between the DMI of Holstein cows and crossbred cows. For *Bos taurus* and crossbred heifers, Oliveira and Ferreira (2016) [5] developed and evaluated models for animals raised under tropical conditions using BW^0.75^ and average daily gain (ADG) as predictors, and this model was evaluated in our study.

Moreover, previous studies have reported that the DMI model proposed by the Dairy NRC (2001) [9] was not adequate for estimating the DMI of replacement heifers [5,11]. Since 2016, additional studies have been published reporting the DMI of heifers raised under different climate, diet, and management conditions. Model evaluation using an updated database is important to ensure that current models adopted by dairy nutritionists adequately represent the actual feed intake of heifers. Gain in genetic merit for milk production may change both feed intake and feed efficiency over time. Moreover, model evaluation is an important approach for identifying whether a new model needs to be updated or developed to estimate the actual DMI better. Therefore, we hypothesized that the current DMI models proposed for Holstein heifers are not adequate for *Bos taurus* or crossbred dairy heifers raised under tropical conditions. The objective of this study was to evaluate previously published DMI equations for heifers using updated data from both *Bos taurus* and crossbred heifers managed under a tropical climate.

## 2. Materials and Methods

### 2.1. Database

The meta-analysis used data from publications indexed in PubMed, SciELO, Web of Science, ScienceDirect, Google Scholar, and Master or Ph.D. theses available online. Terms used for searching the publications were “crossbred”, “dairy heifers”, “dry matter intake”, “Holstein”, “Jersey”, “nutrition”, “tropical”, and “young cattle”. The inclusion criteria were as follows: (1) studies published between 1998 and 2020 (comprising 22 years), (2) data from heifers before first calving (lactation number = 0); (3) post-weaning animals, (4) description data for both dietary ingredients and animal descriptions (BW, ADG, and breed), (5) data for observed intake or estimated DMI of grazing heifers using internal or external markers, (6) studies performed under a tropical climate (regions between Tropic of Capricorn and Tropic of Cancer), (7) measurement data for individual DMI for confined animals (no pen-based measurements), and (8) experiments conducted with *ad libitum* intake. The ADG value was obtained directly from published documents. It was not calculated. It was either a full or a shrunk ADG, depending on how the study was performed. The remaining scientific publications in the final dataset must fit the above-mentioned criteria, and present most of the searched terms in their text content.

The final database was developed using data from 61 studies (*n* = 230 treatment means) of dairy heifers (*n* = 1513 heifers). A list of the selected studies is provided in Supplementary Material S1 (Table S1 [13,14,15,16,17,18,19,20,21,22,23,24,25,26,27,28,29,30,31,32,33,34,35,36,37,38,39,40,41,42,43,44,45,46,47,48,49,50,51,52,53,54,55,56,57,58,59,60,61,62,63,64,65,66,67,68,69,70,71,72,73,74,75]). Animals were grouped based on their genotypes as follows: (1) *Bos taurus* (Holstein, Jersey, Brown Swiss, and Holstein × Jersey) or (2) crossbred *Bos taurus* × *Bos indicus* (Holstein × Gyr, Holstein × Boran, and others Holstein × Zebu). As an exploratory step, a mixed model was used to verify the difference in DMI between these formed groups, including the fixed effects of group, ADG, BW, and the random effects of the study. A significant difference (*p* = 0.0239, *Bos taurus* = 6.7 kg/d vs. Crossbred = 6.2 kg/d) was found and the grouping was maintained.

### 2.2. Calculations

Dietary composition was calculated using the proportion of ingredients in the diet and their nutritional composition when dietary composition was not provided in the study. When heifers were subjected to fasting, BW was calculated as BW/0.96 [9] (Table 1).

If total digestible nutrients (TDN) were not reported, it was calculated from the apparent digestibility coefficient of dry matter (ADDM) or neutral detergent fiber (NDF) using equations published by Cappelle et al. (2001) [76] (Table 1). The energy partitioning of the diet was estimated using the Dairy NRC (2001) [9] model.

### 2.3. Models Evaluated

The DMI of dairy heifers was estimated using the seven equations (Table 1). Hoffman et al. (2008) [11] proposed equations for Holstein (Model 1, HH) and crossbred Holstein × Jersey dairy heifers (Model 2, HHJ). These nonlinear models included the BW as an independent variable. The linear model described by Quigley et al. (1986a) [7] (Model 3, QUI) used BW, BW^0.75^, TDN, ADG, and their interactions as predictor variables. Stallings et al. (1985) [6] (Model 4, STA) used BW as an independent variable in a quadratic polynomial regression model. The Dairy NRC (2001) [9] equation (Model 5, NRC) included BW^0.75^ and net energy for maintenance (NE_m_) to predict the DMI of dairy heifers. Oliveira and Ferreira (2016) [5] proposed two DMI equations for crossbred heifers (*Bos taurus* × *Bos indicus*) raised under Brazilian tropical conditions. These equations were developed using linear (Model 6, OFLin) and nonlinear (Model 7, OFNLin) models, where ADG and BW^0.75^ were included as independent variables.

### 2.4. Statistical Analysis

Descriptive analyses were conducted using SAS (MEANS, REG, and UNIVARIATE procedures; SAS, 2012 [77]). Preliminary plots and model evaluations were performed using R (version 3.5.1; R Development Core Team, 2018 [78]). 

The models were evaluated for both accuracy and precision using the significance of the mean bias and slope bias, in addition to the mean square prediction error (MSPE) and its decomposition (% of the error related to the dispersion, slope, and mean bias) [79]. The concordance correlation coefficient (CCC) [80] and goodness of fit (R^2^) were also calculated. The root means square error (RMSE)—observations standard deviation ratio (RSR) was also used to evaluate the models, which incorporates the benefits of error index statistics, varying from the optimal value of 0 to a large positive value, with zero RSR being a perfect model simulation [81]. The most important statistics considered to rank the performance of models were as follows: 1st, the *p*-values of mean and slope bias, 2nd—MSPE values and their decomposition, 3rd—mean bias, and 4th—when necessary, other models evaluation statistics were used for comparison.

## 3. Results

### 3.1. Database

A total of 61 studies (230 treatment means from 1513 dairy replacement heifers) published as scientific articles (70%, *n* = 1089 heifers), Master’s thesis (21%, *n* = 314 heifers), Ph.D. thesis (7%, *n* = 86 heifers), and proceedings of annual meetings (2%, *n* = 24 heifers) were used (Table 2). These studies were conducted in Brazil (82%, *n* = 1201 heifers), Mexico (7%, *n* = 105 heifers), Ethiopia (5%, *n* = 94 heifers), Kenya (5%, *n* = 97 heifers), and Thailand (1%, *n* = 16 heifers).

Most of these studies used crossbred dairy heifers (67%, *n* = 1167 heifers), followed by *Bos taurus* (26%, *n* = 346 heifers), and only four studies used both (7%) (Table 2). In terms of feeding systems, the heifers were raised in confinement (79%, *n* = 1185 heifers), exclusively pasture (10%, *n* = 168 heifers), and pasture with concentrate supplementation (11%, *n* = 160 heifers). Estimated DMI with internal or external markers was performed in pasture-based studies (21%). Data on animal performance, characteristics, and dietary composition are presented in Table 3.

Studies presented a similar median age and BW at the beginning of the experiments (*Bos taurus*: age = 356 d and BW = 181 kg; crossbred: age = 397 d and BW = 198 kg; Table 3). Crossbred dairy heifers had a similar median DMI as *Bos taurus* (6.2 versus 6.5 kg/d) when raised under tropical conditions. Additionally, diets of *Bos taurus* and crossbred heifers contained similar median levels of forage (approximately 70%) and energy content (digestible energy (DE), metabolizable energy (ME), net energy for maintenance (NE_m_), and net energy for growth (NE_g_)) (Table 3).

### 3.2. Model Evaluation 1: Bos taurus Dairy Heifers

**Figure 1:**
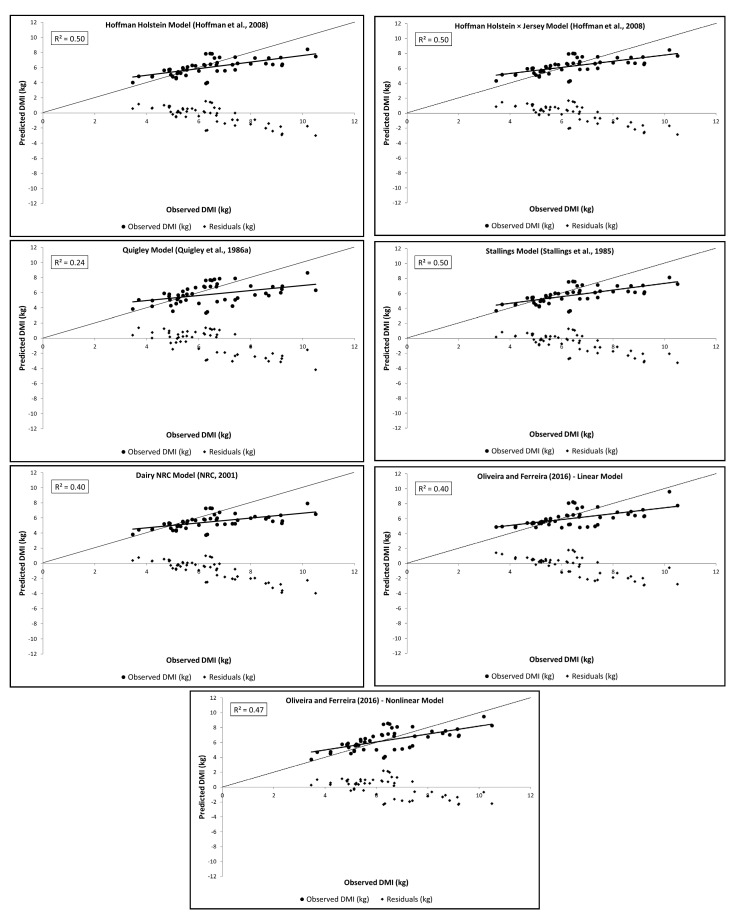
Observed versus model-predicted values and residuals versus model-predicted values from the prediction equations of DMI of *Bos taurus* dairy heifers (*n* = 56).

A total of 56 treatment means (*n* = 20 studies) were used to evaluate the DMI equations for *Bos taurus* dairy heifers. The average heifer DMI was 6.5 kg/d (Table 3). Plots of the observed, predicted, and residual values are shown in Figure 1. The observed DMI was overestimated at low levels and underestimated at high levels for all the equations.

The STA (0.50), HHJ (0.50), and HH (0.50) equations presented the highest R^2^, followed by the OFNLin (0.47) equation. The other equations presented an R^2^ ≤ 0.40 (Table 4). Lower MSPE values were obtained with HHJ, HH, and OFNLin equations (≤1.25 kg^2^/d^2^), whereas in the other equations, the MSPE values were ≥1.35 kg^2^/d^2^ (Table 4). For MSPE decomposition, the dispersion or random error provided the greatest contribution to the MSPE values of all models evaluated (>63%), with higher values for HHJ and OFNLin (>93%). However, for NRC (*p* = 0.0001), STA (*p* = 0.0011), QUI (*p* = 0.0015), HH (*p* = 0.0141), and OFLin models (*p* = 0.0463), the mean bias significantly contributed to the MSPE (36%, 26%, 16%, 10%, and 7%, respectively) (Table 4). Only the QUI model (*p* = 0.0143) presented a significant slope bias. The HHJ and OFNLin models did not present significance for either mean or slope bias. A lower mean bias was found for the OFNLin and HHJ equations (0.13 and 0.16 kg/d, respectively), whereas other equations had a mean bias ≥ 0.36 kg/d, varying by more than 5% of the mean DMI. In contrast, we found a lower slope bias value for the STA (0.04), NRC (0.05), and HH (0.06) equations. The HHJ (0.71), HH (0.74), and OFNLin (0.75) models had lower RSR values than the other models (≥0.81). The highest CCC value was detected for the OFNLin equation (0.67), whereas all other models had CCC values ≤ 0.63.

### 3.3. Model Evaluation 2: Crossbred Dairy Heifers (Bos taurus × Bos indicus)

**Figure 2:**
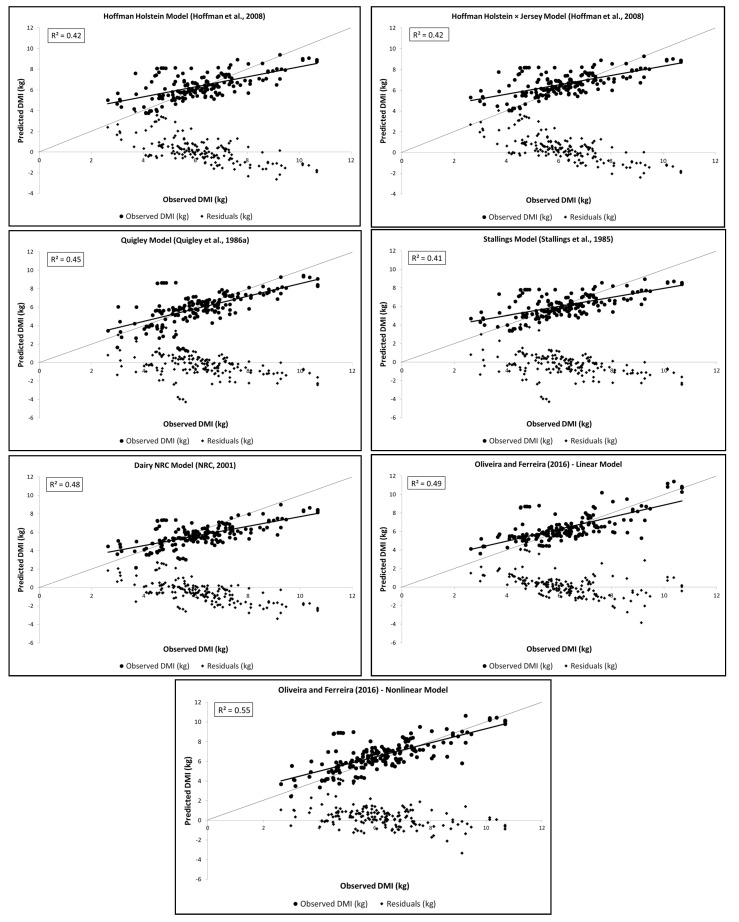
Observed versus model-predicted values and residuals versus model-predicted values from the prediction equations of DMI of dairy heifers *Bos taurus* × *Bos indicus* (*n* = 174).

A total of 174 treatment means (*n* = 45 studies) were used to evaluate the DMI of crossbred dairy heifers. The average heifer DMI was 6.2 kg/d (Table 3). Plots of the observed, predicted, and residual values are shown in Figure 2. The observed DMI was overestimated at low levels and underestimated at high levels for all the equations.

The OFNLin equation presented the highest R^2^ (0.55), and all other models had R^2^ values ≤ 0.49. Lower MSPE values were obtained for OFNLin (1.20 kg^2^/d^2^), OFLin (1.22 kg^2^/d^2^), STA (1.25 kg^2^/d^2^), NRC (1.26 kg^2^/d^2^), and HH (1.26 kg^2^/d^2^), whereas the other equations had MSPE values ≥ 1.30 kg^2^/d^2^ (Table 5). For MSPE decomposition, the dispersion or random error provided the greatest contribution to the MSPE values of all models evaluated (>79%), with higher values for STA and HH (>95%). The STA model was the only one that did not present a significant mean (*p* = 0.3293) or slope bias (*p* = 0.0542). All the other models presented a significant mean bias (*p* < 0.05), resulting in a considerable contribution of the mean bias to MSPE. For QUI, OFLin, and OFNLin models (*p* = 0.0001 for all), the slope bias significantly contributed to the MSPE (17%, 9%, and 9%, respectively) (Table 5).

The STA, HH, OFLin, and QUI equations presented lower mean biases (0.09, −0.20, −0.20, and 0.22 kg/d, respectively), whereas the others varied by more than 5% of mean DMI (Table 5). A lower slope bias occurred for HHJ, NRC, HH, and STA (−0.08, −0.08, −0.14, and −0.15 kg/d, respectively), whereas the slope bias was ≤−0.23 kg/d for other equations. This evaluation showed that the HHJ, HH, and NRC models also did not present a significant slope bias (*p* > 0.05), in addition to the STA equation (Table 5). The RSR values were similar among models, with OFNLin (0.74) presenting the lowest value and QUI (0.83) the highest (Table 5). The OFNLin had a higher CCC value (0.72) than the other equations evaluated in this study (CCC ≤ 0.69), but this model had a significant mean (*p* = 0.0001) and slope bias (*p* = 0.0001) (Table 5).

## 4. Discussion

We evaluated seven equations used to predict the DMI of *Bos taurus* and crossbred (*Bos taurus* × *Bos indicus*) dairy heifers raised in tropical conditions. We focused on evaluating existing models instead of developing new equations because there are already numerous DMI equations developed for dairy heifers in the literature. Although all models evaluated in this study could be used to predict DMI of dairy heifers, our results indicated that the HHJ and OFNLin equations were the most accurate and precise for *Bos taurus* heifers because they did not present significant slope or mean bias and, also, presented lower MSPE and mean bias values. The QUI, STA, NRC, HH, and OFLin models presented significant mean bias and higher values of MSPE, resulting in low precision and accuracy, respectively. All equations evaluated using the *Bos taurus* data underestimated the DMI.

The STA equation was the most adequate DMI model for crossbred heifers because it had the lowest mean bias and did not present significant slope or mean bias. All other crossbred models had a significant mean bias, whereas QUI, OFLin, and OFNLin also presented significant slope bias associated with a lower DMI for crossbred cattle than Holstein cattle. Both OFNLin and OFLin presented significant slope and mean biases, despite OFNLin having the highest precision (R^2^ = 0.55), which contributed to a high CCC. All the equations overestimated the DMI of crossbred heifers, except for the models proposed by QUI, STA, and NRC. The OFNLin and HHJ for *Bos taurus* and HH and STA for crossbreeds showed a higher error because of the disturbance than other models, indicating that random variation was not associated with a correlation among predictors or unknown parameters, which is desirable. 

As was presented, the observed DMI was overestimated at low levels and underestimated at high levels for all the equations for both datasets. It can be an effect of BW as a predictive variable present in all the equations. A lower proportion of data from heavier and lighter heifers could have affected the models’ evaluation at those points. Another explanation could be a difference in the concentrate-to-forage ratio of the diets for younger (from 3 until 10–12 months of age) and older heifers (from 12 months of age until calving) [82]. Older heifers receive less concentrate in the diet than younger heifers because of their lower energy needs and greater intake potential [82]. It results in chemostatic and physical feed intake limitation for younger and older heifers, respectively. That effect of different diet compositions may not be captured by the DMI predictive models, causing over and underestimation for younger and older heifers, respectively.

Substantial differences between *Bos taurus* and crossbred dairy cattle have been described in previous studies, such as heat stress tolerance, milk yield, milk composition, and milking ease [83,84,85]. However, our results showed that the DMI of crossbred heifers raised in tropical conditions might be affected by similar factors to those previously described for *Bos taurus* heifers, especially BW. Moreover, another study reported no differences in NE_m_ requirements between Holstein and crossbred dairy heifers [86].

Oliveira and Ferreira (2016) [5] developed equations to predict the DMI of crossbred dairy heifers raised in Brazilian tropical conditions, and we evaluated these models. Similar to that study, our database was developed using data from studies previously published in the literature, but we used additional studies available from 2016 to 2020. Although some articles in the database were similar to our database, these models showed a significant slope bias for crossbred heifers. This fact could be partially explained by the large number of observations from different countries used in our study, and our database was separated into two subsets of data based on animal genetics (*Bos taurus* and crossbred). Therefore, the main differences between our study and those of Oliveira and Ferreira (2016) [5] are related to the dataset (37 treatment means from 11 studies versus 230 treatment means from 61 studies in our database), the number of observations used for model evaluation (from 21 to 37 observations versus 58 *Bos taurus* and 174 crossbred observations used in our evaluation), and calf sex (female and male calves versus only female data used in our study), and we also evaluated the model proposed by Stallings et al. (1985) [6]. Interestingly, OFNLin performed well for *Bos taurus* instead of crossbred heifers. This could be related to the development dataset of the model used by Oliveira and Ferreira (2016) [5], which had 21 treatment means (37%) from *Bos taurus*, thereby explaining the better performance of OFNLin for those cattle. For crossbred heifers, a possible reparameterization or factor adjustment of the OFLin and OFNLin models could help solve the problem and improve the prediction of these equations, especially the OFNLin model, which presented the highest R^2^ and lowest MSPE.

The low accuracy and precision of QUI (for *Bos taurus*) and NRC equations could be related to multicollinearity problems, such as BW and BW^0.75^ as independent variables in the model [87]. The variance inflation index (VIF) is usually used to identify multicollinearity among variables, where values higher than 5 could indicate multicollinearity [88]. If the VIF values are inflated, the model estimates are less reliable. When a model has multicollinearity, the standard error values of the regression coefficients are increased, the predicted values are biased, and the predictors are not significant even with a high R^2^ [89]. Therefore, the low adequacy of the NRC equation could be associated with multicollinearity problems for variables with high correlation, such as NE_m_ and BW^0.75^. Hoffman et al. (2008) [11] and Oliveira and Ferreira (2016) [5] also reported a similar problem when NRC equation was evaluated using an independent database.

The DMI values found in our study agreed with those reported by the Dairy NRC (2001) [9] (~6.0 kg/d); however, the use of different feed ingredients in Brazil and the United States could result in different feed digestibility values. This value is used to calculate TDN, DE, ME, and consequently, NE_m_, which is an input in the Dairy NRC model. Thus, the TDN value may be a major source of variation, explaining the mean bias for the NRC and QUI models. Moreover, the DMI equation adopted by the Dairy NRC (2001) [9] was developed using beef cattle data [10], which explains its low adequacy for dairy heifers. Dairy cattle breeds have a higher proportion of internal organs (liver, intestine, and kidney) than beef cattle [90,91], which is related to higher NE_m_ requirements. On the other hand, heifers present lower NE_m_ requirements compared with bulls (~15%) [92]. The NE_m_ requirements for dairy heifers were estimated to be 86 kcal/kg of SBW^0.75^ (shrunk body weight) [9,93]. However, a study suggested that NE_m_ requirements of heifers increased over time, similar to cows [94], and estimated as 122 kcal/kg of SBW^0.75^ for Holstein heifers from 6 to 22 months of age [95]. For crossbred Holstein × Gyr heifers, the requirement of NE_m_ is suggested to be between 104 kcal/kg of SBW^0.75^ (17 months and ^1^/_2_Holstein × ^1^/_2_Gyr) [86] and 67 kcal/kg of SBW^0.75^ (3 to 7 months and ^1^/_2_Holstein × ^1^/_2_Gyr) [96]. We used BW, SBW, and EBW for the comparisons previously described. For Jiao et al. (2015) [95] and Moreira (2016) [86], we used SBW = 0.96 × BW [9] because in that study the NE_m_ requirement values were expressed as BW^0.75^, but for Castro et al. (2020) [96], we used EBW = 0.894 × SBW because the NE_m_ requirement values were originally expressed as EBW^0.75^. Therefore, the low accuracy of predictions from the DMI model of NRC heifers could also be related to differences in NE_m_ requirements and genetic variation in crossbred heifers. Scarce data regarding NE_m_ requirements for Holstein × Zebu cattle have been published.

Although important studies on the nutrient requirements of crossbred dairy cattle (*Bos taurus* × *Bos indicus*) have been published [96,97,98], data on heifers are still scarce. Since 2016, heifer studies have been performed to better understand the effects of ADG and supply of nutrients on mammary gland development in prepubertal and pubertal crossbred heifers [69,99,100]. Moreover, other studies have evaluated the effects of gestation days on the body and conceptus composition and nutrient use by pregnant crossbred cows [101,102]. 

It is important to note that Dairy NRC (2001) [9] suggested adjustments in DMI for pregnant heifers based on days of pregnancy (DP). An adjustment for DP (AdjDMI) > 210 to <259 d can be calculated using the following equation: AdjDMI = (1 + ((210 − DP) × 0.0025)), where DP is the days in pregnancy. For DP ≥ 259 d, the Dairy NRC (2001) [9] suggested using a different equation to estimate the DMI of heifers, DMI = (1.71 − (0.69 × e^((0.35 × DP − 280))))/100 × BW, where e is the Euler number (e = 2.718), DP is the number of days in the pregnancy, and BW is the body weight. However, these equations were not evaluated in the present study. The DMI decreases around calving based on factors related to the animal (DP and body condition score (BCS)) and diet (contents of NDF, ether extract, and rumen undegradable protein) [103]. Feeding programs can affect DMI around calving, resulting in changes in blood metabolites and body composition of crossbred cows [101,102].

Although our objective was not to evaluate the predicted DMI of heifers around calving, nonlinear models may more adequately describe the DMI reduction around calving for dairy heifers, such as that fitted by Hoffman et al. (2008) [11]. A previous equation was developed using data from Holstein heifers [2] during the transition period, considering DMI = 1.713 − 0.688 × e^((0.344 × DP)), where DP = days in pregnancy. Limited information on DMI in pregnant dairy heifers is available. Heifers with similar gestation lengths but different ages, BCS, dietary management, and feed efficiency have different levels of DMI [103,104].

The STA equation (mean bias = 0.09/kg/d and MSPE = 1.25 kg^2^/d^2^) was the most adequate in predicting the DMI of crossbred dairy heifers raised under tropical conditions. However, only a few studies have evaluated this equation. Studies performed by Hoffman et al. (2008) [11] and Oliveira and Ferreira (2016) [5] did not evaluate the STA equation. The STA, HH, and HHJ models included only BW as a predictor, whereas the OFNLin and OFLin models also use ADG as a predictor; thus, these models could be used when diet information is unavailable. However, it is known that dietary factors such as NDF and TDN affect DMI in dairy heifers [105]. Additionally, our results showed that heifers are usually fed a high-roughage diet (approximately 70% for both *Bos taurus* and crossbred heifers). It is known that the roughage-to-concentrate ratio affects feed intake, where high-forage diets with poor fiber digestibility can reduce the DMI by a physical limitation, although diets with high contents of TDN (mainly from starch and lipids) cause a chemostatic intake regulation [106]. Furthermore, diets with a high proportion of fiber resulted in a lower passage rate and lower feed efficiency in dairy heifers (kg of body weight gain/kg of feed intake). However, high dietary fiber content limits the DMI and reduces the energy content of the diet, which is desirable for heifers over 12 months [82]. A few models to predict DMI of heifers from dietary components have been reported, but they require more inputs [5,7,11], which may limit their use.

Factors related to the environment can also affect feed intake in heifers, among which air temperature (AT) is the major factor. The DMI can decrease considerably in extreme AT and is common with high ATs during hot seasons. Nonaka et al. (2008) [4] found a decrease of 9% and 8% in DMI at 33 °C compared to 20 °C and 28 °C, respectively, for prepubertal Holstein heifers kept under constant relative humidity (60%), suggesting that heifer DMI decreased under 28 °C. Limited data are available for other environmental variables that might influence DMI in heifers, but previous studies have been performed such investigations using lactating cows [107]. 

Independent variables related to the environment could improve the accuracy of DMI estimates in dairy heifers, such as environmental AT. When Quigley et al. (1986b) [108] studied the factors that could affect DMI in dairy heifers, AT was evaluated as a predictive variable and, despite the effect of AT on DMI being statistically significant, they concluded that this variable did not improve MSPE and R^2^. However, that research did not include data from animals subjected to high temperatures because the AT range was from −11.3 °C to 25.6 °C. Oliveira and Ferreira (2016) [5] also evaluated AT as an independent variable in the DMI model, but the data were limited. Linear equations were developed by Hoffman et al. (2008) [11], including AT, but these equations were less accurate than nonlinear models. Another possibility could be the use of the temperature-humidity index (THI) as a predictor to improve the model adequacy of these equations. For dairy cattle, heat stress can be considered as occurring at THI > 68 when milk yield is critically reduced [109]. Above this THI limit, DMI, milk yield, and milk fat and protein yields decreased considerably [110].

We still have opportunities to improve models for predicting DMI of dairy heifers using dietary and/or environmental factors besides BW. Models using dietary factors as inputs have been developed, but their small improvements in model accuracy have discouraged their use [5,11]. It occurs especially because a DMI model is applied before the diet formulation and values of dietary variables are not known at this moment. The use of THI as a predictor in DMI models is interesting, especially in tropical countries where heifers are raised on pastures and are susceptible to stressful environmental conditions. Moreover, this could be useful for the summer season in humid subtropical climates. However, it is necessary to consider that different values of relative humidity and AT could result in similar THI values, and many times, it does not include other climatic variables in its calculation [111], which can result in bias. Additionally, there is an opportunity to improve our DMI models for heifers using data from electronic sensors and machine learning approaches, which could help dairy nutritionists to meet the nutrient requirements of heifers in different feeding programs.

## 5. Conclusions

For *Bos taurus* dairy heifers raised in tropical conditions the models developed by Hoffman et al. (2008) [11], especially the HHJ model, and the non-linear model developed by Oliveira and Ferreira (2016) [5] were the most suitable for DMI prediction, whereas for crossbred heifers, the most suitable was the Stallings et al. (1985) [6] model. In general, the Holstein model developed by Hoffman et al. (2008) [11] and the Dairy NRC (2001) model [9] had a significant mean bias, resulting in lower precision, whereas OFLin had a significant slope bias, resulting in lower accuracy, and the QUI model presented both. There is evidence that models from *Bos taurus* heifers could be used to estimate the DMI of crossbred heifers under tropical conditions. Dietary and environmental factors can affect the DMI of dairy heifers, but additional evaluation using multimodel or machine learning approaches should be performed using a larger database. Future equations should consider different nutritional plans, dietary compositions, and environmental conditions during the growing period. Equations based especially on machine learning with big data could improve the predictive power of the DMI models.

## Figures and Tables

**Table 1:** Empirical equations used in the development of the study.

Description	Unity	Equation
SBW ^1^	kg	BW × 0.96
TDN ^2^	% DM	−3.84 + 1.064 × ADDM
TDN ^3^	% DM	91.0246 − 0.571588 × NDF
DE ^1^	Mcal	TDN × 0.04409
ME ^1^	Mcal	1.01 × DE − 0.45
NE_m_ ^1^	Mcal	$1.37\;\times \;\mathrm{ME}\;-\;0.138{\;\times \;\mathrm{ME}}^{2}\;+\;0.0105{\;\times \;\mathrm{ME}}^{3\;}-\;1.12$
NE_g_ ^1^	Mcal	$1.42\;\times \;\mathrm{ME}\;-\;0.174{\;\times \;\mathrm{ME}}^{2}+0.0122{\;\times \;\mathrm{ME}}^{3}\;-\;1.65$
Intake prediction equations		
HH	kg DM/day	$15.36{\times (1-e}^{(-0.0022\times \mathrm{BW})})$
HHJ	kg DM/day	$12.91{\times (1-e}^{(-0.00295\times \mathrm{BW})})$
QUI	kg DM/day	$-29.86\;-\;0.0000154{\;\times \;\mathrm{BW}}^{2}+0.157{\;\times \;\mathrm{BW}}^{0.75}+2.09\;\times \;\mathrm{ADG}\;-\;0.118{\;\times \;\mathrm{ADG}}^{2}+0.73\times \mathrm{TDN}$ $-0.0048{\;\times \;\mathrm{TDN}}^{2}-0.0014\;\times \;\mathrm{BW}\;\times \;\mathrm{ADG}\;-\;0.019\;\times \;\mathrm{TDN}\;\times \;\mathrm{ADG}$
STA	kg DM/day	$-0.417+0.03325\;\times \;\mathrm{BW}\;-\;0.0000266154{\;\times \;\mathrm{BW}}^{2}$
NRC	kg DM/day	$({\left(\mathrm{SBW}\right)}^{0.75\;}\times \;(0.2435{\;\times \;\mathrm{NE}}_{m}-0.0466{\;\times \;\mathrm{NE}}_{m}{}^{2}-0.1128{))/\mathrm{NE}}_{m}$
OFLin	kg DM/day	$8.7147-0.2402{\;\times \;\mathrm{BW}}^{0.75}+0.0027{\;\times \;(\mathrm{BW}}^{0.75}{)}^{2}+3.6050\;\times \;\mathrm{ADG}\;-\;1.4168{\;\times \;\mathrm{ADG}}^{2}$
OFNLin	kg DM/day	$0.1175{\;\times \;\mathrm{BW}}^{0.75}-3.4984{\;\times \;e}^{(-2.4690\times \mathrm{ADG})}$

^1^ Equations from Dairy NRC (2001) [9]; ^2^ TDN prediction equation based on the apparent digestibility of dry matter [76]; ^3^ TDN prediction equation based on the neutral detergent fiber in the diet [76]; SBW = shrunk body weight; TDN = total digestible nutrients in the diet; DE = digestible energy; ME = metabolizable energy; NE_m_ = net energy for maintenance; NE_g_ = net energy for growth; HH = dry matter intake equation for Holstein heifers from Hoffman et al. (2008) [11]; HHJ = dry matter intake equation for crossbred Holstein × Jersey heifers from Hoffman et al. (2008) [11]; QUI = dry matter intake equation for dairy heifers from Quigley et al. (1986a) [7]; STA = dry matter intake equation for dairy heifers from Stallings et al. (1985) [6]; NRC = dry matter intake equation for dairy heifers from Dairy NRC (2001) [9]; OFLin = linear dry matter intake equation from Oliveira and Ferreira (2016) [5]; OFNLin = nonlinear dry matter intake equation from Oliveira and Ferreira (2016) [5]; BW = body weight, ADDM = apparent digestibility of the dry matter from the diet (%); NDF = neutral detergent fiber of the diet (% DM); BW = live body weight (kg); ADG = average daily gain (kg).

**Table 2:** Summary on the number of studies, observations, and heifers for each breed, rearing system, type of publication, and country.

Variable	No. Studies	%	No. Treatment Means	%	No. Heifers	%
Breed						
*Bos taurus*	16	26.2	56	25.0	346	22.9
Crossbred ^1^	41	67.2	174	75.0	1167	77.1
Both	4	6.6	–	–	–	–
Rearing system						
Confinement	48	78.7	189	82.2	1185	78.3
Pasture	6	9.8	8	3.5	168	11.1
Pasture + Supplement	7	11.5	33	14.3	160	10.6
Type of publication						
Scientific paper	43	70.5	170	73.9	1089	72.0
Congress paper	1	1.6	2	0.9	24	1.6
Master’s thesis	13	21.3	43	18.7	314	20.8
Ph.D.’s thesis	4	6.6	15	6.5	86	5.6
Country						
Brazil	50	81.9	188	81.7	1201	79.4
Ethiopia	3	5.0	14	6.1	94	6.2
Mexico	4	6.5	13	5.7	105	6.9
Kenya	3	5.0	11	4.8	97	6.4
Thailand	1	1.6	4	1.7	16	1.1
Total	61	100.0	230	100.0	1513	100.0

^1^ Crossbred Bos taurus × Bos indicus.

**Table 3:** Descriptive statistical analyses for the variables in the database related to experimental planning, heifer characteristics, and the diets provided to *Bos taurus* (*n* = 56) and crossbred *Bos taurus* × *Bos indicus* dairy heifers (*n* = 174).

Variables	Experimental and Animal Variables							
	*Bos taurus*				Crossbred			
	*n*	Mean	Median	Range	*n*	Mean	Median	Range
Period of adaptation (days)	30	17	15	10–45	121	19	15	10–45
Experimental period (days)	56	80	84	56–120	166	84	84	84–180
Fasting (hours)	28	14.13	16.00	12.00–16.00	68	13.56	14.00	12.00–16.00
Age (days)	40	295	356	90–512	113	358	397	107–702
Initial BW (kg)	56	197.25	181.00	58.70–403.83	174	213.15	198.30	75.72–412.83
Mean BW (kg)	56	234.74	238.41	133.85–424.79	174	249.02	241.50	127.84–430.07
Final BW (kg)	56	263.39	256.11	155.00–445.75	174	277.10	274.65	161.94–447.31
ADG (kg)	56	0.77	0.79	0.24–1.21	174	0.75	0.82	−0.03–1.40
FCE (kg/kg)	20	9.27	7.69	5.78–16.67	84	7.78	6.64	4.01–21.35
DMI (kg DM/day)	56	6.53	6.30	3.46–10.50	174	6.20	6.03	2.63–10.68
**Variables**	**Dietary factors**							
	***Bos taurus***				**Crossbred**			
	***n***	**Mean**	**Median**	**Range**	***n***	**Mean**	**Median**	**Range**
% of Roughage (%)	56	73.01	71.20	22.37–100.00	161	69.56	70.00	20.41–100.00
DM (%) *	36	45.71	40.45	12.30–90.51	144	54.46	48.97	12.38–92.47
OM (% DM)	42	91.20	93.15	79.60–97.90	121	92.55	92.74	86.04–97.40
CP (% DM)	47	14.26	14.20	7.98–22.63	164	13.76	13.78	7.06–22.10
NDF (% DM)	47	45.30	44.10	29.40–65.60	158	50.29	46.27	23.17–88.10
ADF (% DM)	32	26.84	27.10	16.80–34.70	119	26.50	23.93	15.39–45.00
EE (% DM)	18	2.14	1.62	0.80–5.53	114	2.52	2.40	0.80–6.75
TCHO (% DM)	10	81.67	81.29	76.68–86.03	41	77.59	77.01	72.20–88.35
NFC (% DM)	17	34.66	36.30	18.03–44.20	89	31.84	33.42	7.90–50.74
MM (% DM)	42	8.82	6.85	2.10–20.40	121	7.45	7.26	2.60–13.96
TDN (% DM)	56	64.79	63.96	52.60–81.87	174	63.96	65.42	42.70–76.28
ADDM (%)	31	65.82	66.08	52.25–80.55	114	64.57	65.00	22.50–75.61
DE (Mcal/kg)	56	2.86	2.82	2.32–3.61	174	2.82	2.88	1.88–3.36
ME (Mcal/kg)	56	2.43	2.40	1.89–3.20	174	2.40	2.46	1.45–2.95
NE_m_ (Mcal/kg)	56	1.54	1.52	1.05–2.19	174	1.51	1.57	0.61–1.99
NE_g_ (Mcal/kg)	56	0.94	0.92	0.50–1.51	174	0.91	0.97	0.08–1.34

* *Bos taurus*: minimum of 12.30% of DM = diet based on only fresh sweet potato vines; and crossbred: minimum of 12.38% of DM = diet with 60% inclusion of fresh forage cactus (*Opuntia ficus-indica* Mill), which constituted 9.28% of the DM; N = number of observations; BW = live body weight; ADG = average daily gain; FCE = feed conversion efficiency; DMI = dry matter intake; DM = dietary dry matter; OM = dietary organic matter; CP = dietary crude protein; NDF = dietary neutral detergent fiber; ADF = dietary acid detergent fiber; EE = dietary ether extract; TCHO = dietary total carbohydrates; NFC = dietary non-fibrous carbohydrates; MM = dietary mineral matter; TDN = dietary total digestible nutrients; ADDM = dietary apparent digestibility of dry matter; DE = dietary digestible energy; ME = dietary metabolizable energy; NE_m_ = dietary net energy for maintenance; NE_g_ = dietary net energy for growth.

**Table 4:** Evaluation statistics of the dry matter consumption equations of dairy heifers *Bos taurus* (*n* = 56).

Statistics	Observed	HH	HHJ	QUI	STA	NRC	OFLin	OFNLin
DMI	6.53	6.13	6.36	5.84	5.84	5.55	6.17	6.39
R²	‒	0.50	0.50	0.24	0.50	0.40	0.40	0.47
MSPE	‒	1.24	1.19	1.68	1.36	1.61	1.35	1.25
MSPE, % mean	‒	19.03	18.28	25.71	20.88	24.74	20.75	19.14
MSPE decomposition (%)								
Mean Bias, % MSE	‒	10.47	1.90	16.81	25.77	36.36	7.03	1.14
Slope Bias, % MSE	‒	0.23	1.09	8.82	0.11	0.14	3.43	5.45
Dispersion, % MSE	‒	89.30	97.01	74.36	74.12	63.50	89.54	93.40
Mean Bias	‒	0.40	0.16	0.69	0.69	0.97	0.36	0.13
Slope Bias	‒	0.05	0.12	−0.38	0.04	0.06	−0.19	−0.20
P-Mean Bias	‒	0.0141	0.3061	0.0015	0.0001	0.0001	0.0463	0.4283
P-Slope Bias	‒	0.7079	0.4389	0.0143	0.7797	0.7352	0.1560	0.0815
RSR	‒	0.74	0.71	1.00	0.82	0.97	0.81	0.75
CCC	‒	0.63	0.63	0.43	0.59	0.44	0.60	0.67

DMI = dry matter intake; R^2^ = coefficient of determination; MSPE = mean squared prediction error; MSE = mean squared error; RSR = RMSE-observations standard deviation ratio; CCC = concordance correlation coefficient; HH = DMI equation for Holstein dairy heifers from Hoffman et al. (2008) [11]; HHJ = DMI equation for Holstein × Jersey dairy heifers from Hoffman et al. (2008) [11]; QUI = DMI equation for dairy heifers from Quigley et al. (1986a) [7]; STA = DMI equation for dairy heifers from Stallings et al. (1985) [6]; NRC = DMI equation for dairy heifers from Dairy NRC (2001) [9]; OFLin = linear dry matter intake equation from Oliveira and Ferreira (2016) [5]; OFNLin = nonlinear dry matter intake equation from Oliveira and Ferreira (2016) [5].

**Table 5:** Evaluation statistics of the dry matter consumption equations of crossbred dairy heifers *Bos taurus* × *Bos indicus* (*n* = 174).

Statistics	Observed	HH	HHJ	QUI	STA	NRC	OFLin	OFNLin
DMI	6.20	6.39	6.61	5.97	6.11	5.71	6.40	6.59
R²	‒	0.42	0.42	0.45	0.41	0.48	0.49	0.55
MSPE	‒	1.26	1.30	1.34	1.25	1.26	1.22	1.20
MSPE, % mean	‒	20.26	21.00	21.65	20.16	20.28	19.76	19.34
MSPE decomposition (%)								
Mean Bias, % MSE	‒	2.44	10.08	2.78	0.55	14.85	2.70	10.93
Slope Bias, % MSE	‒	1.79	0.47	17.58	2.13	0.53	8.95	8.75
Dispersion, % MSE	‒	95.77	89.45	79.64	97.32	84.61	88.35	80.32
Mean Bias	‒	−0.20	−0.41	0.22	0.09	0.48	−0.20	−0.40
Slope Bias	‒	−0.14	−0.08	−0.34	−0.15	−0.08	−0.25	−0.23
P-Mean Bias	‒	0.0392	0.0001	0.0275	0.3293	0.0001	0.0298	0.0001
P-Slope Bias	‒	0.0744	0.3428	0.0001	0.0542	0.2987	0.0001	0.0001
RSR	‒	0.78	0.81	0.83	0.77	0.78	0.76	0.74
CCC	‒	0.62	0.58	0.66	0.62	0.63	0.69	0.72

DMI = dry matter intake; R^2^ = coefficient of determination; MSPE = mean squared prediction error; MSE = mean squared error; RSR = RMSE-observations standard deviation ratio; CCC = concordance correlation coefficient; HH = DMI equation for Holstein dairy heifers from Hoffman et al. (2008) [11]; HHJ = DMI equation for Holstein × Jersey dairy heifers from Hoffman et al. (2008) [11]; QUI = DMI equation for dairy heifers from Quigley et al. (1986a) [7]; STA = DMI equation for dairy heifers from Stallings et al. (1985) [6]; NRC = DMI equation for dairy heifers from Dairy NRC (2001) [9]; OFLin = linear dry matter intake equation from Oliveira and Ferreira (2016) [5]; OFNLin = nonlinear dry matter intake equation from Oliveira and Ferreira (2016) [5].

## Supplementary Materials

The following are available online at https://www.mdpi.com/article/10.3390/ani11113181/s1, Table S1: Selected studies to create a database for the evaluation of dry matter intake equations for replacement dairy heifers.

## References

[1] Bareille N., Beaudeau F., Billon S., Robert A., Faverdin P. (2003). Effects of health disorders on feed intake and milk production in dairy cows. Livest. Prod. Sci..

[2] Hayirli A., Grummer R.R., Nordheim E.V., Crump P.M. (2003). Models for predicting dry matter intake of Holsteins during the prefresh transition period. J. Dairy Sci..

[3] Grummer R.R., Mashek D.G., Hayirli A. (2004). Dry matter intake and energy balance in the transition period. Vet. Clin. N. Am. Food. Anim. Pr..

[4] Nonaka I., Takusari N., Tajima K., Suzuki T., Higuchi K., Kurihara M. (2008). Effects of high environmental temperatures on physiological and nutritional status of prepubertal Holstein heifers. Livest. Sci..

[5] Oliveira A.S., Ferreira V.B. (2016). Prediction of intake in growing dairy heifers under tropical conditions. J. Dairy. Sci..

[6] Stallings C.C., Kroll G., Kelley J.C., McGilliard M.L. (1985). A computer ration evaluation program for heifers, dry cows, and lactating cows. J. Dairy Sci..

[7] Quigley J.D., James R.E., McGilliard M.L. (1986). Dry matter intake in dairy heifers. 2. Equations to predict intake of heifers under intensive management. J. Dairy Sci..

[8] National Research Council (NRC) (1989). Nutrient Requirements of Dairy Cattle.

[9] National Research Council (NRC) (2001). Nutrient Requirements of Dairy Cattle.

[10] National Research Council (NRC) (1996). Nutrient Requirements of Beef Cattle.

[11] Hoffman P.C., Weigel K.A., Wernberg R.M. (2008). Evaluation of equations to predict dry matter intake of dairy heifers. J. Dairy Sci..

[12] Souza V.L. (2015). Parameterization and Evaluation of Models to Estimate the Requirements and Performance of Dairy Cattle for Use in Brazil. Ph.D. Thesis.

[13] Aguiar M.S.M.A., Silva F.F., Donato S.L.R., Rodrigues E.S.O., Costa L.T., Mateus R.G., Souza D.R., Silva V.L. (2015). Forage cactus in diets of confined dairy cattle: Performance and economic viability. Semina Ciênc. Agrár..

[14] Alemu T., Chairatanayuth P., Vijchulata P., Tudsri S. (2005). The potential of urea treated maize stover for growth performance of weaned crossbred calves. Kasetsart. J..

[15] Almeida G.A.P., Campos J.M.S., Ferreira M.A., Correia A.L.V., Andrade A.P. (2015). Palm (*Opuntia ficus indica* mill) cv. Giant in supplements for growth dairy females in pasture. Rev. Caatinga..

[16] Aranda E., Mendoza G.D., García-Bojalil C., Castrejón F. (2001). Growth of heifers grazing stargrass complemented with sugar cane, urea and a protein supplement. Livest. Prod. Sci..

[17] Araújo W.A., Paulino P.V., Marcondes M.I., Carvalho C.G.V., Silva F.C.O. (2011). Performance and carcass traits of crossbred heifers from three genetic groups fed corn or sorghum silage based diets. Ciência Anim. Bras..

[18] Barbosa L.S. (2012). Uso de Sombreamento Sobre índices Térmicos, Respostas Fisiológicas e Desempenho de Bezerras Cruzadas ½ Holandês × ½ Jersey a Pasto. Master’s Thesis.

[19] Barros L.J.A., Ferreira M.A., Oliveira J.C.V., Santos D.C., Chagas J.C.C., Alves A.M.S.V., Silva A.E.M., Freitas W.R. (2018). Replacement of Tifton hay by spineless cactus in Girolando post-weaned heifers’ diets. Trop Anim. Health Prod..

[20] Burgos E.M.G. (2013). Desempenho de Novilhas Leiteiras Alimentadas com Diferentes Volumosos. Master’s Thesis.

[21] Carvalho M.C., Ferreira M.A., Cavalcanti C.V.A., Lima L.E., Silva F.M., Miranda K.F., Véras A.S.C., Azevedo M., Vieira V.C.F. (2005). Association of sugar cane bagasse, forage cactus and urea with different supplements in diets of Holstein heifers. Acta Sci. Anim. Sci..

[22] Coronel-Robles U., Ortega-Cerrilla M.E., Mendoza-Martínez G.D., Zetina-Córdoba P., Torres-Esqueda M.T.S., Munguía-Ameca G., Teco-Jácome M.V. (2016). Productive response and progesterone concentration in Holstein heifers supplemented with Saccharomyces cerevisiae1077 or Saccharomyces boulardii1079. J. Anim. Plant Sci..

[23] Cruz A.A.C. (2018). Desempenho de Novilhas Girolando Alimentadas com Dietas à Base de Palma Forrageira, Cana-de-Açúcar Mais ureia e Concentrado. Master’s Thesis.

[24] Dias A.M., Silva F.F., Veloso C.M., Ítavo L.C.V., Pires A.J.V., Souza D.R., Sá J.F., Mendes F.B.L., Nascimento P.V.N. (2008). Cassava bagasse in diets of dairy heifers: Intake of nutrients and productive performance. Arq. Bras. Med. Vet. Zootec..

[25] Faria E.S. (2000). Avaliação de Níveis de Fibra na Dieta de Novilhas Leiteiras de Diferentes Grupos Zootécnicos. Doctor’s Thesis.

[26] Farias M.S., Prado I.N., Valero M.V., Zawadzki F., Silva R.R., Eiras C.E., Rivaroli D.C., Lima B.S. (2012). Glycerine levels for crossbred heifers growing in pasture: Performance, feed intake, feed efficiency and digestibility. Semin. Ciênc. Agrár..

[27] Franco M.O., Marcondes M.I., Campos J.M.S., Detmann E., Valadares Filho S.C., Freitas D.R. (2016). Performance of dairy females fed dried yeast from sugar cane. Acta Sci. Anim. Sci..

[28] Gallo P.C.S., Pereira M.N., Campos G.P., Gallo S.B. (2019). Effects of neutral detergent fiber concentration of sugarcane-based diets on the performance of Holstein heifers. Semin. Ciênc. Agrár..

[29] Garcia J.A.S., Vieira P.F., Cecon P.R., Setti M.C., McManus C., Louvandini H. (2006). Performance of growing cattle fed sunflower meal. Ciência Anim. Bras..

[30] Gojjam Y., Tolera A., Mesfin R. (2011). Management options to accelerate growth rate and reduce age at first calving in Friesian-Boran crossbred heifers. Trop Anim. Health Prod..

[31] Gonçalves M.F., Oliveira M.V., Nogueira H.C.R., Santos A.P.S., França A.M.S., Hermisdorff I.C., Santos R.M. (2014). Desempenho de novilhas alimentadas com co-produtos da indústria do milho ou do ácido cítrico. Vet. Not..

[32] Guimarães A.V. (2010). Desempenho de Novilhas Leiteiras Alimentadas com Farelo de Mamona e Valor Energético do Farelo e Torta da Mamona. Master’s Thesis.

[33] Inácio J.G. (2016). Bagaço de Cana-de-Açúcar Como Volumoso Exclusivo Para Novilhas Leiteiras. Master’s Thesis.

[34] Jenet A., Fernandez-Rivera A., Tegegne A., Yimegnuhal A., Osuji P.O., Kreuzer M. (2004). Growth and feed conversion of Boran (*Bos indicus*) and Holstein × Boran heifers during three physiological states receiving different levels of a tropical diet. Livest Prod. Sci..

[35] Kaitho R.J., Kariuki J.N. (1998). Effects of Desmodium, Sesbania and Calliandra supplementation on growth of dairy heifers fed Napier grass basal diet. Asian-Austral J. Anim. Sci..

[36] Kamphayae S., Kumagai H., Butcha P., Ritruechai V., Udchachon S. (2017). Yeast mixture of liquid beer and cassava pulp with rice straw for the growth of dairy heifers. Trop Anim. Health Prod..

[37] Kariuki J.N., Gachuiri C.K., Gitau G.K., Tamminga S., Van Bruchem J., Muia J.M.K., Irungu K.R.G. (1998). Effect of feeding Napier grass, lucerne and sweet potato vines as sole diets to dairy heifers on nutrient intake, weight gain and rumen degradation. Livest Prod. Sci..

[38] Kariuki J.N., Gitau G.K., Gachuiri C.K., Tamminga S., Muia J.M.K. (1999). Effect of supplementing napier grass with desmodium and lucerne on DM, CP and NDF intake and weight gains in dairy heifers. Livest Prod. Sci..

[39] Lage C.F.S. (2016). Desenvolvimento Corporal, Idade à Puberdade e Desenvolvimento da Glândula Mamária de Fêmeas Mestiças Leiteiras Aleitadas com Diferentes Teores de Sólidos Totais na Dieta Líquida. Master’s Thesis.

[40] Lima M.L.M., Fernandes J.J.R., Carvalho E.R., Santos S.C., Cruz M.C., Brito A.C.F. (2009). Performance of dairy crossbred heifers fed sugar cane corrected and supplemented with concentrate having Quillaja saponaria molina extract. Ciência Anim. Bras..

[41] Machado A.F., Guimarães S.E.F., Guimarães J.D., Santos G.M., Silva A.L., Silva Y.F.R.S., Netto D.S.L., Correa P.V.F., Marcondes M.I. (2020). Effect of protein supplement level on the productive and reproductive parameters of replacement heifers managed in intensive grazing systems. PLoS ONE.

[42] Maciel R.P., Neiva J.N.M., Araujo V.L., Cunha O.F.R., Paiva J., Restle J., Mendes C.Q., Lôbo R.N.B. (2012). Intake, nutrient digestibility and performance of dairy heifers fed diets containing palm kernel cake. Rev. Bras. Zootec..

[43] Martins P.C. (2017). Consumo Alimentar Residual e Ganho de Peso Residual em Novilhas f1 Girolando. Master’s Thesis.

[44] Matos B.C. (2009). Efeito da Relação Proteína Metabolizável: Energia Metabolizável da Ração de Novilhas Pré-Púberes em Crescimento Acelerado. Master’s Thesis.

[45] Mendes Neto J., Campos J.M.S., Valadares Filho S.C., Lana R.P., Queiroz A.C., Euclydes R.F. (2007). Effects of partial replacement of Tifton 85 hay with citrus pulp on intake, performance, and development of dairy heifers. Rev. Bras. Zootec..

[46] Mendonça B.P.C., Lana R.P., Mancio A.B., Detmann E., Barbosa A.M., Guimarães G. (2010). Levels of mineral mixture and urea in supplementation of crossbred heifers, with Gyr predominance, reared at pasture during the dry season. Rev. Bras Zootec..

[47] Miranda L.F., Queiroz A.C., Valadares Filho S.C., Cecon P.R., Pereira E.S., Paulino M.F., Campos J.M.S., Miranda J.R. (1999). Performance and ponderal development of dairy heifers fed sugar cane-based diets. Rev. Bras. Zootec..

[48] Miranda L.F., Queiroz A.C., Valadares Filho S.C., Cecon P.R., Pereira E.S., Campos J.M.S., Lana R.P., Miranda J.R. (1999). Ingestive behavior of dairy heifers fed sugar cane based diets. Rev. Bras. Zootec..

[49] Molina-Botero I.C., Arroyave-Jaramillo J., Valencia-Salazar S., Barahona-Rosales R., Aguilar-Pérez C.F., Burgos A.A., Arango J., Ku-Vera J.C. (2019). Effects of tannins and saponins contained in foliage of Gliricidia sepium and pods of Enterolobium cyclocarpum on fermentation, methane emissions and rumen microbial population in crossbred heifers. Anim. Feed Sci. Technol..

[50] Monteiro C.C.F., Melo A.A.S., Ferreira M.A., Campos J.M.S., Souza J.S.R., Silva E.T.S., Andrade R.P.X., Silva E.C. (2014). Replacement of wheat bran with spineless cactus (*Opuntia ficus indica* Mill cv Gigante) and urea in the diets of Holstein × Gyr heifers. Trop Anim. Health Prod..

[51] Mora B.V., Castillo-Gallegos E., Alonso-Díaz M.Á., Ocanã-Zavaleta E., Jarillo-Rodríguez J. (2017). Live-weight gains of Holstein × Zebu heifers grazing a Cratylia argentea/Toledo-grass (Brachiaria brizantha) association in the Mexican humid tropics. Agroforest Syst..

[52] Mota D.A., Berchielli T.T., Canesin R.C., Rosa B.L., Ribeiro A.F., Brandt H.V. (2013). Nutrient intake, productive performance and body measurements of dairy heifers fed with different sources of protein. Acta Sci. Anim. Sci..

[53] Oliveira M.V.M., Lana R.P., Freitas A.W.P., Eifert E.C., Pereira J.C., Valadares Filho S.C., Pérez J.R.O. (2005). Effects of different dietary levels of monensin on nutrient digestibility and on ruminal, blood and urinary metabolites in dairy heifers. Rev. Bras. Zootec..

[54] Oliveira M.V.M., Lana R.P., Eifert E.C., Luz D.F., Vargas Junior F.M. (2009). Performance of Holstein heifers in feedlot receiving monensin at different levels. Rev. Bras. Zootec..

[55] Ornelas L.T.C., Silva D.C., Tomich T.R., Campos M.M., Machado F.S., Ferreira A.L., Maurício R.M., Pereira L.G.R. (2019). Differences in methane production, yield and intensity and its effects on metabolism of dairy heifers. Sci. Total Environ..

[56] Pancoti C.G. (2019). Exigências Nutricionais de Energia em Novilhas Gir, Holandês e F1—Holandês × Gir. Doctor’s Thesis.

[57] Pereira J.C., Silva P.R.C., Cecon P.R., Resende Filho M.A., Oliveira R.L. (2003). Broiler-litter and supplement based on ruminal microbiota in dairy heifers diets: Performance and economic evaluation. Rev. Bras. Zootec..

[58] Pereira J.C., Cunha D.N.F.V., Cecon P.R., Faria E.S. (2008). Performance, rectal temperature and respiratory ratio of dairy heifers from three genetic groups fed diets with different levels of fiber. Rev. Bras. Zootec..

[59] Pinheiro A.A., Veloso C.M., Rocha Neto A.L., Silva R.R., Silva F.F., Mendes F.B.L., Santana Júnior H.A., Azevedo S.T., Carvalho G.G.P. (2012). Ingestive behavior of dairy heifers fed cocoa (“Theobroma cacao”) meal levels in the diet. Rev. Bras. Saúde Prod. Anim..

[60] Queiroz M.F.S. (2010). Teores Crescentes de Proteína Bruta em Dietas à Base de Cana-de-Açúcar Para Novilhas Holandês × Gir. Doctor’s Thesis.

[61] Quirino D.F. (2019). Behavior, Performance, and Tick Incidence in Girolando and Holstein Grazing Heifers. Master’s thesis.

[62] Rangel A.H.N., Campos J.M.S., Oliveira A.S., Valadares Filho S.C., Assis A.J., Souza S.M. (2010). Performance and nutritional parameters of growing heifers fed corn silage or sugar cane with concentrate. Rev. Bras. Zootec..

[63] Rodrigues A.A., Flores O.S., Ferreira Junior A.G., Netto D.P., Ferreira R.P., Pedroso A.F. (2009). Dry matter intake and weight gain of dairy heifers fed sugar cane and grazing alfalfa. 46ª Reunião Anual da Sociedade Brasileira de Zootecnia.

[64] Santana D.F.Y., Lira M.A., Santos M.V.F., Ferreira M.A., Santos D.C., Mello A.C.L., Dubeux Júnior J.C.B., Araujo G.G.L. (2010). Dry matter intake and performance of Girolando and Guzerá heifers and Guzerá under supplementation in caatinga, during the rainy season, in Pernambuco, Brazil. Rev. Bras. Zootec..

[65] Santos S.A., Campos J.M.S., Valadares Filho S.C., Detmann E., Oliveira A.S., Souza S.M. (2010). Productive performance of growing dairy heifers fed corn silage and soybean or cottonseed meal based concentrate. Rev. Bras. Zootec..

[66] Siécola Júnior S., Bitencourt L.L., Melo L.Q., Silveira V.A., Lopes N.M., Silva J.R.M., Pereira R.A.N., Pereira M.N. (2014). Deleafed sugarcane and performance of heifers and dairy cows. Arq. Bras. Med. Vet. Zootec..

[67] Silva F.F., Aguiar M.S.M.A., Veloso C.M., Pires A.J.V., Bonomo P., Dutra G.S., Almeida V.S., Carvalho G.G.P., Silva R.R., Dias A.M. (2006). Performance of dairy heifers fed on elephantgrass silage added with different levels of cassava bagasse. Arq. Bras. Med. Vet. Zootec..

[68] Silva D.C. (2017). Metabolismo em Novilhas Girolando Com Fenótipos Divergentes Para Eficiência Alimentar. Master’s Thesis.

[69] Silva A.L., Detmann E., Dijkstra J., Pedroso A.M., Silva L.H.P., Machado A.F., Sousa F.C., Santos G.B., Marcondes M.I. (2018). Effects of rumen-undegradable protein on intake, performance, and mammary gland development in prepubertal and pubertal dairy heifers. J. Dairy Sci..

[70] Sousa M.G. (2018). Proteína Degradável no Rúmen em Suplementos Múltiplos Para Novilhas GIROLANDAS à Pasto. Master’s Thesis.

[71] Souza A.L., Garcia R., Bernardino F.S., Campos J.M.S., Valadares Filho S.C., Cabral L.S., Gobbi K.F. (2006). Coffee hulls in dairy heifers diet: Intake, digestibility, and production. Rev. Bras. Zootec..

[72] Souza D.D. (2018). Farelo de Mamona em Dietas para Novilhas Leiteiras Em Pastejo. Doctor’s Thesis.

[73] Teixeira R.M.A., Campos J.M.S., Valadares Filho S.C., Oliveira A.S., Assis A.J., Pina D.S. (2007). Intake, digestibility and performance of dairy heifers fed coffee hulls replacing of corn silage. Rev. Bras. Zootec..

[74] Teixeira F.A., Silva F.F., Bonomo P., Pires A.J.V., Nascimento P.V.N., Gonçalves Neto J. (2014). Performance of dairy heifers grazing on Urochloa decumbens pastures deferred for two periods. Acta Sci. Anim. Sci..

[75] Vieira V.C.F. (2006). Associação do Bagaço de Cana-de-açúcar, Palma Forrageira e Ureia com Diferentes Suplementos em Dietas de Novilhas da raça HOLANDESA. Master’s Thesis.

[76] Cappelle E.R., Valadares Filho S.C., Silva J.F.C., Cecon P.R. (2001). Estimates of the energy value from chemical characteristics of the feedstuffs. Rev. Bras. Zootec..

[77] SAS Institute, Inc (2012). SAS OnDemand for Academics. Release 9.04.01M5P09132017.

[78] R Development Core Team (2018). R: A Language and Environment for Statistical Computing. Version 3.1.1..

[79] Bibby J., Toutenburg H. (1977). Prediction and Improved Estimation in Linear Models.

[80] Lin L. (1989). A concordance correlation coefficient to evaluate reproducibility. Biometrics.

[81] Moriasi D.N., Arnold J.G., Van Liew M.W., Bingner R.L., Harmel R.D., Veith T.L. (2007). Model evaluation guidelines for sys-tematic quantification of accuracy in watershed simulations. Trans. ASABE.

[82] Erickson P.S., Anderson J.L., Kalscheur K.F., Lascano G.J., Akins M.S., Heinrichs A.J. (2020). Symposium review: Strategies to improve the efficiency and profitability of heifer raising. J. Dairy Sci..

[83] Negrão J.A., Marnet P. (2006). Milk yield, residual milk, oxytocin and cortisol release during machine milking in Gir, Gir × Holstein and Holstein cows. Reprod. Nutr. Dev..

[84] Alfonzo E.P.M., Silva M.V.G.B., Daltro D.S., Stumpf M.T., Dalcin V.C., Kolling G., Fischer V., McManus C.M. (2016). Relationship between physical attributes and heat stress in dairy cattle from different genetic groups. Int. J. Biometeorol..

[85] Ludovico A., Trentin M., Rêgo F.C.A. (2019). Sources of variation of dairy production and milk composition in Holstein cows, Jersey, and Girolando. Arch. De Zootec..

[86] Moreira T.S. (2016). Energy Requirements, Energetic Partition and Methane Emission from Growing Holstein, Gyr and F1 HOLSTEIN-Gyr Dairy Heifers. Ph.D. Thesis.

[87] Grewal R., Cote J.A., Baumgartner H. (2004). Multicollinearity and measurement error in structural equation models: Implications for theory testing. Mark. Sci..

[88] Cohen J., Cohen P., West S.G., Aiken L.S. (1983). Applied Multiple Regression/Correlation Analysis for the Behavioral Sciences.

[89] O’Brien M.R. (2007). A caution regarding rules of thumb for variance inflation factors. Qual. Quant..

[90] Terry C.A., Knapp R.H., Edwards J.W., Mies W.L., Savell J.W., Cross H.R. (1990). Yields of by-products from different cattle types. J. Anim. Sci..

[91] Casas A., Cianzio D., Rivera A. (1997). Comparison of Holstein, Charbray, and Zebu bulls for beef production under rotational grazing II. Offal components and carcass composition. J. Agric. Univ. Puerto Rico.

[92] National Research Council (NRC) (2016). Nutrient Requirements of Beef Cattle.

[93] Fox D.G., Tylutki T.P. (1998). Accounting for the effects of environment on the nutrient requirements of dairy cattle. J. Dairy Sci..

[94] Moraes L.E., Kebreab E., Strathe A.B., Dijkstra J., France J., Casper D.P., Fadel J.G. (2015). Multivariate and univariate analysis of energy balance data from lactating dairy cows. J. Dairy Sci..

[95] Jiao H.P., Yan T., Wills D.A., McDowell D.A. (2015). Maintenance energy requirements of young Holstein cattle from calorimetric measurements at 6, 12, 18, and 22 months of age. Livest. Sci..

[96] Castro M.M.D., Albino R.L., Rodrigues J.P.P., Sguizzato A.L.L., Santos M.M.F., Rotta P.P., Caton J.S., Moraes L.E.F.D., Silva F.F., Marcondes M.I. (2020). Energy and protein requirements of Holstein × Gyr crossbred heifers. Animal.

[97] Oss D.B., Machado F.S., Tomich T.R., Pereira L.G.R., Campos M.M., Castro M.M.D., Silva T.E., Marcondes M.I. (2017). Energy and protein requirements of crossbred (Holstein × Gyr) growing bulls. J. Dairy Sci..

[98] Silva A.L., DeVries T.J., Fernandes E.C., Marcondes M.I. (2020). Development and evaluation of equations to predict growth of Holstein dairy heifers in a tropical climate. J. Dairy Sci..

[99] Weller M.M.D.C.A., Albino R.L., Marcondes M.I., Silva W., Daniels K.M., Campos M.M., Duarte M.S., Mescouto M.L., Silva F.F., Guimarães S.E.F. (2016). Effects of nutrient intake level on mammary parenchyma growth and gene expression in crossbred (Holstein × Gyr) prepubertal heifers. J. Dairy Sci..

[100] Albino R.L., Sguizzato A.L., Daniels K.M., Duarte M.S., Lopes M.M., Guimarães S.E.F., Weller M.M.D.C.A., Marcondes M.I. (2017). Performance strategies affect mammary gland development in prepubertal heifers. J. Dairy Sci..

[101] Rotta P.P., Valadares Filho S.C., Gionbelli T.R.S., Silva L.F.C., Engle T.E., Marcondes M.I., Machado F.S., Villadiego F.A.C., Silva L.H.R. (2015). Effects of day of gestation and feeding regimen in Holstein × Gyr cows: I. Apparent total-tract digestibility, nitrogen balance, and fat deposition. J. Dairy Sci..

[102] Sguizzato A.L.L., Marcondes M.I., Valadares Filho S.C., Caton J., Neville T.L., Machado F.S., Pacheco M.V.C., Rotta P.P. (2020). Body composition changes of crossbred Holstein × Gyr cows and conceptus during pregnancy. J. Dairy Sci..

[103] Hayirli A., Grummer R.R., Nordheim E.V., Crump P.M. (2002). Animal and dietary factors affecting feed intake during the prefresh transition period in Holsteins. J. Dairy Sci..

[104] Korver S., Van Eekelen E.A.M., Vos H., Nieuwhof G.J., Van Arendonk J.A.M. (1991). Genetic parameters for feed intake and feed efficiency in growing dairy heifers. Livest. Prod. Sci..

[105] Tomlinson D.J., James R.E., McGilliard E.D. (1991). Effect of varying levels of neutral detergent fiber and total digestible nutrients on intake and growth of Holstein heifers. J. Dairy Sci..

[106] Van Soest P.J. (1994). Nutritional Ecology of the Ruminant.

[107] West J.W., Mullinix B.G., Bernard J.K. (2003). Effects of hot, humid weather on milk temperature, dry matter intake, and milk yield of lactating dairy cows. J. Dairy Sci..

[108] Quigley J.D., James R.E., McGilliard M.L. (1986). Dry matter intake in dairy heifers. 1. Factors affecting intake of heifers under intensive management. J. Dairy Sci..

[109] Zimbelman R.B., Rhoads R.P., Rhoads M.L., Duff G.C., Baumgard L.H., Collier R.J. (2009). A re-evaluation of the impact of temperature humidity index (THI) and black globe humidity index (BGHI) on milk production in high producing dairy cows. Proceedings of the 22nd Southwest Nutrition and Management Conference.

[110] Gantner V., Mijić P., Kuterovac K., Solić D., Gantner R. (2011). Temperature-humidity index values and their significance on the daily production of dairy cattle. Mljekarstvo.

[111] Nienaber J.A., Hahn G.L. (2007). Livestock production system management responses to thermal challenges. Int. J. Biometeorol..

