# Supplementary material for: Feed Intake of Growing Dairy Heifers Raised under Tropical Conditions: A Model Evaluation Using Meta-Analysis
Source: Animals (Basel). 2021 Nov 7;11(11):3181. doi: 10.3390/ani11113181 (PMC8614301; doi:10.3390/ani11113181)
Supplement: Supplementary file 1 [file animals-11-03181-s001.zip › animals-1368052-supplementary.pdf]

Feed intake of growing dairy heifers raised under tropical conditions: A model evaluation using meta-analysis

M. Busanello, D.G. Sousa, F.A.C. Mendonça, V.L. Daley, R. Almeida, C.M.M. Bittar, and D.P.D. Lanna

Supplementary Material S1

Supplementary Table S1. Selected studies to create a database for the evaluation of dry matter intake equations for replacement dairy heifers.

Authors	Country	N[1]	Breed
Aguiar et al. (2015) [13]	Brazil	4	Crossbred (Holstein × Zebu)
Alemu et al. (2005) [14]	Ethiopia	3	Crossbred (Holstein × Boran)
Almeida et al. (2015) [15]	Brazil	4	Crossbred (Holstein × Zebu)
Aranda et al. (2001) [16]	Mexico	4	Crossbred (Holstein × Zebu)
Araujo et al. (2011) [17]	Brazil	5	Crossbred (Holstein × Zebu)
Barbosa (2012) [18]	Brazil	2	Crossbred (Holstein × Jersey)
Barros et al. (2018) [19]	Brazil	4	Crossbred (Holstein × Gyr)
Burgos (2013) [20]	Brazil	2	Holstein
Carvalho et al. (2005) [21]	Brazil	3	Holstein
Coronel-Robles et al. (2016) [22]	Mexico	3	Holstein
Cruz et al. (2020) [23]	Brazil	4	Crossbred (Holstein × Gyr)
Dias et al. (2008) [24]	Brazil	4	Crossbred (Holstein × Gyr)
Faria (2000) [25]	Brazil	5	Holstein and Crossbred (Holstein × Zebu)
Farias et al. (2012) [26]	Brazil	4	Crossbred (Holstein × Zebu)
Franco et al. (2016) [27]	Brazil	6	Holstein and Crossbred (Holstein × Zebu)
Gallo et al. (2019) [28]	Brazil	3	Holstein
Garcia et al. (2006) [29]	Brazil	4	Holstein
Gojjam et al. (2011) [30]	Ethiopia	2	Crossbred (Holstein × Boran)
Gonçalves et al. (2014) [31]	Brazil	4	Crossbred (Holstein × Zebu)
Guimarães (2010) [32]	Brazil	4	Holstein
Inácio et al. (2017) [33]	Brazil	4	Crossbred (Holstein × Gyr)
Janet et al. (2004) [34]	Ethiopia	9	Crossbred (Holstein × Boran)
Kaitho and Kariuki (1998) [35]	Kenya	4	Holstein

Kamphayae et al. (2017) [36]	Thailand	4	Crossbred (Holstein × Zebu)
Kariuki et al. (1998) [37]	Kenya	3	Holstein
Kariuki et al. (1999) [38]	Kenya	4	Holstein
Lage (2016) [39]	Brazil	4	Crossbred (Holstein × Gyr)
Lima et al. (2009) [40]	Brazil	3	Crossbred (Holstein × Jersey)
Machado et al. (2020) [41]	Brazil	3	Crossbred (Holstein × Gyr)
Maciel et al. (2012) [42]	Brazil	4	Crossbred (Holstein × Zebu)
Martins (2017) [43]	Brazil	4	Crossbred (Holstein × Gyr)
Matos (2009) [44]	Brazil	2	Crossbred (Holstein × Jersey)
Mendes Neto et al. (2007) [45]	Brazil	4	Crossbred (Holstein × Zebu)
Mendonça et al. (2010) [46]	Brazil	5	Crossbred (Holstein × Zebu)
Miranda et al. (1999ab) [47,48]	Brazil	5	Crossbred (Holstein × Zebu)
Molina-Botero et al. (2019) [49]	Mexico	4	Crossbred (Holstein × Zebu)
Monteiro et al. (2014) [50]	Brazil	4	Crossbred (Holstein × Gyr)
Mora et al. (2017) [51]	Mexico	2	Crossbred (Holstein × Zebu)
Mota et al. (2013) [52]	Brazil	4	Crossbred (Holstein × Zebu)
Oliveira et al. (2005; 2009) [53,54]	Brazil	4	Crossbred (Holstein × Zebu)
Ornelas et al. (2019) [55]	Brazil	6	Crossbred (Holstein × Gyr)
Pancoti (2015) [56]	Brazil	2	Holstein and Crossbred (Holstein × Gyr)
Pereira et al. (2003) [57]	Brazil	4	Crossbred (Holstein × Zebu)
Pereira et al. (2008) [58]	Brazil	2	Crossbred (Holstein × Zebu)
Pinheiro et al. (2012) [59]	Brazil	4	Crossbred (Holstein × Gyr)
Queiroz (2010) [60]	Brazil	4	Crossbred (Holstein × Gyr)
Quirino (2019) [61]	Brazil	2	Holstein and Crossbred (Holstein × Gyr)
Rangel et al. (2010) [62]	Brazil	4	Holstein and Brown Swiss
Rodrigues et al. (2009) [63]	Brazil	2	Crossbred (Holstein × Jersey)
Santana et al. (2010) [64]	Brazil	5	Crossbred (Holstein × Gyr)
Santos et al. (2010) [65]	Brazil	4	Crossbred (Holstein × Zebu)
Siécola Júnior et al. (2014) [66]	Brazil	2	Crossbred (Holstein × Gyr)
Silva et al. (2006) [67]	Brazil	4	Crossbred (Holstein × Zebu)
Silva (2017) [68]	Brazil	6	Crossbred (Holstein × Gyr)
Silva et al. (2018) [69]	Brazil	6	Holstein

Sousa (2018) [70]	Brazil	2	Crossbred (Holstein × Gyr)
Souza et al. (2006) [71]	Brazil	4	Crossbred (Holstein × Zebu)
Souza (2018) [72]	Brazil	4	Crossbred (Holstein × Zebu)
Teixeira et al. (2007) [73]	Brazil	4	Crossbred (Holstein × Zebu)
Teixeira et al. (2014) [74]	Brazil	2	Crossbred (Holstein × Zebu)
Vieira (2006) [75]	Brazil	3	Holstein

[1]Number of treatment means.

References

13. Aguiar, M.S.M.A.; Silva, F.F.; Donato, S.L.R.; Rodrigues, E.S.O.; Costa, L.T.; Mateus, R.G.; Souza, D.R.; Silva, V.L. Forage cactus in diets of confined dairy cattle: performance and economic viability. *Semina: Ciênc Agrár* **2015**, 36, 1013–1030. http://dx.doi.org/10.5433/1679-0359.2015v36n2p1013
14. Alemu, T.; Chairatanayuth, P.; Vijchulata, P.; Tudsri, S. The potential of urea treated maize stover for growth performance of weaned crossbred calves. *Kasetsart J* **2005**, 39, 638–646.
15. Almeida, G.A.P.; Campos, J.M.S.; Ferreira, M.A.; Correia, A.L.V.; Andrade, A.P. Palm (*Opuntia ficus indica* mill) cv. Giant in supplements for growth dairy females in pasture. *Rev Caatinga* **2015**, 28,161–171.
16. Aranda, E.; Mendoza, G.D.; García-Bojalil, C.; Castrejón, F. Growth of heifers grazing stargrass complemented with sugar cane, urea and a protein supplement. *Livest Prod Sci* **2001**, 71, 201–206. http://dx.doi.org/10.1016/S0301-6226(01)00188-9
17. Araújo, W.A.; Paulino, P.V.; Marcondes, M.I.; Carvalho, C.G.V.; Silva, F.C.O. Performance and carcass traits of crossbred heifers from three genetic groups fed corn or sorghum silage based diets. *Ci Anim Bras* **2011**, 12, 101–107.
18. Barbosa, L.S. Uso de sombreamento sobre índices térmicos, respostas fisiológicas e desempenho de bezerras cruzadas ½ Holandês x ½ Jersey a pasto. Master's thesis, Goiás State University, Anápolis, GO, Brazil, 2012.
19. Barros, L.J.A.; Ferreira, M.A.; Oliveira, J.C.V.; Santos, D.C.; Chagas, J.C.C.; Alves, A.M.S.V.; Silva, A.E.M.; Freitas, W.R. Replacement of Tifton hay by spineless cactus in Girolando post-weaned heifers' diets. *Trop Anim Health Prod* **2018**, 50, 149–154. https://doi.org/10.1007/s11250-017-1415-4
20. Burgos, E.M.G. Desempenho de novilhas leiteiras alimentadas com diferentes volumosos. Master's thesis, Federal University of Viçosa, Viçosa, MG, Brazil, 2013.
21. Carvalho, M.C.; Ferreira, M.A.; Cavalcanti, C.V.A.; Lima, L.E.; Silva, F.M.; Miranda, K.F.; Véras, A.S.C.; Azevedo, M.; Vieira, V.C.F. Association of sugar cane bagasse, forage cactus and urea with different supplements in diets of Holstein heifers. *Acta Sci Anim Sci* **2005**, 27, 247–252. https://doi.org/10.4025/actascianimsci.v27i2.1229
22. Coronel-Robles, U.; Ortega-Cerrilla, M.E.; Mendoza-Martínez, G.D.; Zetina-Córdoba, P.; Torres-Esqueda, M.T.S.; Munguía-Ameca, G.; Teco-Jácome, M.V. Productive response and progesterone concentration in Holstein heifers supplemented with *Saccharomyces cerevisiae*[1077] or *Saccharomyces boulardii*[1079]. *J Anim Plant Sci* **2016**, 26, 17–24.

23. Cruz, A.A.C. Desempenho de novilhas Girolando alimentadas com dietas à base de palma forrageira, cana-de-açúcar mais ureia e concentrado. Master's thesis, Federal Rural University of Pernambuco, Recife, PE, Brazil, 2018.
24. Dias, A.M.; Silva, F.F.; Veloso, C.M.; Ítavo, L.C.V.; Pires, A.J.V.; Souza, D.R.; Sá, J.F.; Mendes, F.B.L.; Nascimento, P.V.N. Cassava bagasse in diets of dairy heifers: intake of nutrients and productive performance. *Arq Bras Med Vet Zootec* **2008**, 60, 987–995.
25. Faria, E.S. Avaliação de níveis de fibra na dieta de novilhas leiteiras de diferentes grupos zootécnicos. Doctor's thesis, Federal University of Viçosa, Viçosa, MG, Brazil, 2000.
26. Farias, M.S.; Prado, I.N.; Valero, M.V.; Zawadzki, F.; Silva, R.R.; Eiras, C.E.; Rivaroli, D.C.; Lima, B.S. Glycerine levels for crossbred heifers growing in pasture: performance, feed intake, feed efficiency and digestibility. *Semina: Ciênc Agrár* **2012**, 33, 1177–1188. http://dx.doi.org/10.5433/1679-0359.2012v33n3p1177
27. Franco, M.O.; Marcondes, M.I.; Campos, J.M.S.; Detmann, E.; Valadares Filho, S.C.; Freitas, D.R. Performance of dairy females fed dried yeast from sugar cane. *Acta Sci Anim Sci* **2016**, 38, 205–212. https://doi.org/10.4025/actascianimsci.v38i2.30174
28. Gallo, P.C.S.; Pereira, M.N.; Campos, G.P.; Gallo, S.B. Effects of neutral detergent fiber concentration of sugarcane-based diets on the performance of Holstein heifers. *Semina: Ciênc Agrár* **2019**, 40, 947–956. http://dx.doi.org/10.5433/1679-0359.2019v40n2p947
29. Garcia, J.A.S.; Vieira, P.F.; Cecon, P.R.; Setti, M.C.; McManus, C.; Louvandini, H. Performance of growing cattle fed sunflower meal. *Ci Anim Bras* **2006**, 7, 223–233.
30. Gojjam, Y.; Tolera, A.; Mesfin, R. Management options to accelerate growth rate and reduce age at first calving in Friesian-Boran crossbred heifers. *Trop Anim Health Prod* **2011**, 43, 393–399. https://doi.org/10.1007/s11250-010-9705-0
31. Gonçalves, M.F.; Oliveira, M.V.; Nogueira, H.C.R.; Santos, A.P.S.; França, A.M.S.; Hermisdorff, I.C.; Santos, R.M. Desempenho de novilhas alimentadas com co-produtos da indústria do milho ou do ácido cítrico. *Vet Not* **2014**, 20, 28–36. https://doi.org/10.14393/VTV20N1a2014.24600
32. Guimarães, A.V. Desempenho de novilhas leiteiras alimentadas com farelo de mamona e valor energético do farelo e torta da mamona. Master's thesis, Federal University of Viçosa, Viçosa, MG, Brazil, 2010.
33. Inácio, J.G. Bagaço de cana-de-açúcar como volumoso exclusivo para novilhas leiteiras. Master's thesis, Federal Rural University of Pernambuco, Recife, PE, Brazil, 2016.
34. Jenet, A.; Fernandez-Rivera, A.; Tegegne, A.; Yimegnuhal, A.; Osuji, P.O.; Kreuzer, M. Growth and feed conversion of Boran (*Bos indicus*) and Holstein × Boran heifers during three physiological states receiving different levels of a tropical diet. *Livest Prod Sci* **2004**, 89, 159–173. https://doi.org/10.1016/j.livprodsci.2004.02.005
35. Kaitho, R.J.; Kariuki, J.N. Effects of Desmodium, Sesbania and Calliandra supplementation on growth of dairy heifers fed *Napier* grass basal diet. *Asian-Austral J Anim Sci* **1998**, 11, 680–684. https://doi.org/10.5713/ajas.1998.680
36. Kamphayae, S.; Kumagai, H.; Butcha, P.; Ritruechai, V.; Udchachon, S. Yeast mixture of liquid beer and cassava pulp with rice straw for the growth of dairy heifers. *Trop Anim Health Prod* **2017**, 49, 491–496. https://doi.org/10.1007/s11250-016-1218-z
37. Kariuki, J.N.; Gachuiri, C.K.; Gitau, G.K.; Tamminga, S.; Van Bruchem, J.; Muia, J.M.K.; Irungu, K.R.G. Effect of feeding *Napier* grass, lucerne and sweet potato vines as sole diets to

dairy heifers on nutrient intake, weight gain and rumen degradation. *Livest Prod Sci* **1998**, 55, 13–20. https://doi.org/10.1016/S0301-6226(98)00127-4

38. Kariuki, J.N.; Gitau, G.K.; Gachuiri, C.K.; Tamminga, S.; Muia, J.M.K. Effect of supplementing napier grass with desmodium and lucerne on DM, CP and NDF intake and weight gains in dairy heifers. *Livest Prod Sci* **1999**, 60, 81–88. https://doi.org/10.1016/S0301-6226(99)00035-4
39. Lage, C.F.S. Desenvolvimento corporal, idade à puberdade e desenvolvimento da glândula mamária de fêmeas mestiças leiteiras aleitadas com diferentes teores de sólidos totais na dieta líquida. Master's thesis, Federal University of Minas Gerais, Belo Horizonte, MG, Brazil, 2016.
40. Lima, M.L.M.; Fernandes, J.J.R.; Carvalho, E.R.; Santos, S.C.; Cruz, M.C.; Brito, A.C.F. Performance of dairy crossbred heifers fed sugar cane corrected and supplemented with concentrate having *Quillaja saponaria molina* extract. *Ci Anim Bras* **2009**, 10, 730–734.
41. Machado, A.F.; Guimarães, S.E.F.; Guimarães, J.D.; Santos, G.M.; Silva, A.L.; Silva, Y.F.R.S.; Netto, D.S.L.; Correa, P.V.F.; Marcondes, M.I. Effect of protein supplement level on the productive and reproductive parameters of replacement heifers managed in intensive grazing systems. *Plos One* **2020**, 15, e0239786. https://doi.org/10.1371/journal.pone.0239786
42. Maciel, R.P.; Neiva, J.N.M.; Araujo, V.L.; Cunha, O.F.R.; Paiva, J.; Restle, J.; Mendes, C.Q.; Lôbo, R.N.B. Intake, nutrient digestibility and performance of dairy heifers fed diets containing palm kernel cake. *Rev Bras Zootec* **2012**, 41, 698–706. https://doi.org/10.1590/S1516-35982012000300033
43. Martins, P.C. Consumo alimentar residual e ganho de peso residual em novilhas f1 Girolando. Master's thesis, Federal University of Minas Gerais, Belo Horizonte, MG, Brazil, 2017.
44. Matos, B.C. Efeito da relação proteína metabolizável:energia metabolizável da ração de novilhas pré-púberes em crescimento acelerado. Master's thesis, University of São Paulo, Piracicaba, SP, Brazil, 2009. https://doi.org/10.11606/D.11.2009.tde-09112009-143654
45. Mendes Neto, J.; Campos, J.M.S.; Valadares Filho, S.C.; Lana, R.P.; Queiroz, A.C.; Euclydes, R.F. Effects of partial replacement of *Tifton 85* hay with citrus pulp on intake, performance, and development of dairy heifers. *Rev Bras Zootec* **2007**, 36, 626–634. https://doi.org/10.1590/S1516-35982007000300016
46. Mendonça, B.P.C; Lana, R.P.; Mancio, A.B.; Detmann, E.; Barbosa, A.M.; Guimarães, G. Levels of mineral mixture and urea in supplementation of crossbred heifers, with Gyr predominance, reared at pasture during the dry season. *Rev Bras Zootec* **2010**, 39, 2273–2280. https://doi.org/10.1590/S1516-35982010001000025
47. Miranda, L.F.; Queiroz, A.C.; Valadares Filho, S.C.; Cecon, P.R.; Pereira, E.S.; Paulino, M.F.; Campos, J.M.S.; Miranda, J.R. Performance and ponderal development of dairy heifers fed sugar cane-based diets. *Rev Bras Zootec* **1999a**, 28, 605–613. https://doi.org/10.1590/S1516-35981999000300025
48. Miranda, L.F.; Queiroz, A.C.; Valadares Filho, S.C.; Cecon, P.R.; Pereira, E.S.; Campos, J.M.S.; Lana, R.P.; Miranda, J.R. Ingestive behavior of dairy heifers fed sugar cane based diets. *Rev Bras Zootec* **1999b**, 28, 614–620. https://doi.org/10.1590/S1516-35981999000300026
49. Molina-Botero, I.C.; Arroyave-Jaramillo, J.; Valencia-Salazar, S.; Barahona-Rosales, R.; Aguilar-Pérez, C.F.; Burgos, A.A.; Arango, J.; Ku-Vera, J.C. Effects of tannins and saponins contained in foliage of *Gliricidia sepium* and pods of *Enterolobium cyclocarpum* on fermentation, methane emissions and rumen microbial population in crossbred heifers. *Anim Feed Sci Technol* **2019**, 251, 1–11. https://doi.org/10.1016/j.anifeedsci.2019.01.011

50. Monteiro, C.C.F.; Melo, A.A.S.; Ferreira, M.A.; Campos, J.M.S.; Souza, J.S.R.; Silva, E.T.S.; Andrade, R.P.X.; Silva, E.C. Replacement of wheat bran with spineless cactus (*Opuntia ficus indica* Mill cv Gigante) and urea in the diets of Holstein x Gyr heifers. *Trop Anim Health Prod* **2014**, 46, 1149–1154. https://doi.org/10.1007/s11250-014-0619-0
51. Mora, B.V.; Castillo-Gallegos, E.; Alonso-Díaz, M.Á.; Ocanã-Zavaleta, E.; Jarillo-Rodríguez, J. Live-weight gains of Holstein × Zebu heifers grazing a *Cratylia argentea*/Toledo-grass (*Brachiaria brizantha*) association in the Mexican humid tropics. *Agroforest Syst* **2017**, 91, 1057–1068. https://doi.org/10.1007/s10457-016-9980-5
52. Mota, D.A.; Berchielli, T.T.; Canesin, R.C.; Rosa, B.L.; Ribeiro, A.F.; Brandt, H.V. Nutrient intake, productive performance and body measurements of dairy heifers fed with different sources of protein. *Acta Sci Anim Sci* **2013**, 35, 273–279. https://doi.org/10.4025/actascianimsci.v35i3.18749
53. Oliveira, M.V.M.; Lana, R.P.; Freitas, A.W.P.; Eifert, E.C.; Pereira, J.C.; Valadares Filho, S.C.; Pérez, J.R.O. Effects of different dietary levels of monensin on nutrient digestibility and on ruminal, blood and urinary metabolites in dairy heifers. *Rev Bras Zootec* **2005**, 34, 2143–2154. https://doi.org/10.1590/S1516-35982005000600040
54. Oliveira, M.V.M.; Lana, R.P.; Eifert, E.C.; Luz, D.F.; Vargas Junior, F.M. Performance of Holstein heifers in feedlot receiving monensin at different levels. *Rev Bras Zootec* **2009**, 38,1835–1840. https://doi.org/10.1590/S1516-35982009000900028
55. Ornelas, L.T.C.; Silva, D.C.; Tomich, T.R.; Campos, M.M.; Machado, F.S.; Ferreira, A.L.; Maurício, R.M.; Pereira, L.G.R. Differences in methane production, yield and intensity and its effects on metabolism of dairy heifers. *Sci Total Environ* **2019**, 689, 1133–1140. https://doi.org/10.1016/j.scitotenv.2019.06.489
56. Pancoti, C.G. Exigências nutricionais de energia em novilhas Gir, Holandês e F1 – Holandês x Gir. Doctor's thesis, Federal University of Minas Gerais, Belo Horizonte, MG, Brazil, 2019.
57. Pereira, J.C.; Silva, P.R.C.; Cecon, P.R.; Resende Filho, M.A.; Oliveira, R.L. Broiler-litter and supplement based on ruminal microbiota in dairy heifers diets: Performance and economic evaluation. *Rev Bras Zootec* **2003**, 32, 653–662. https://doi.org/10.1590/S1516-35982003000300017
58. Pereira, J.C.; Cunha, D.N.F.V.; Cecon, P.R.; Faria, E.S. Performance, rectal temperature and respiratory ratio of dairy heifers from three genetic groups fed diets with different levels of fiber. *Rev Bras Zootec* **2008**, 37, 328–334. https://doi.org/10.1590/S1516-35982008000200020
59. Pinheiro, A.A.; Veloso, C.M.; Rocha Neto, A.L.; Silva, R.R.; Silva, F.F.; Mendes, F.B.L.; Santana Júnior, H.A.; Azevedo, S.T.; Carvalho, G.G.P. Ingestive behavior of dairy heifers fed cocoa ("*Theobroma cacao*") meal levels in the diet. *Rev Bras Saúde Prod Anim* **2012**, 13, 224–236. https://doi.org/10.1590/S1519-99402012000100020
60. Queiroz, M.F.S. Teores crescentes de proteína bruta em dietas à base de cana-de-açúcar para novilhas Holandês x Gir. Doctor's thesis, São Paulo State University, Jaboticabal, SP, Brazil, 2010.
61. Quirino, D.F. Behavior, performance, and tick incidence in Girolando and Holstein grazing heifers. Master's thesis, Federal University of Viçosa, Viçosa, MG, Brazil, 2019.
62. Rangel, A.H.N.; Campos, J.M.S.; Oliveira, A.S.; Valadares Filho, S.C.; Assis, A.J.; Souza, S.M. Performance and nutritional parameters of growing heifers fed corn silage or sugar cane with concentrate. *Rev Bras Zootec* **2010**, 39, 2518–2526. https://doi.org/10.1590/S1516-35982010001100027

63. Rodrigues, A.A.; Flores, O.S.; Ferreira Junior, A.G.; Netto, D.P.; Ferreira, R.P.; Pedroso, A.F. Dry matter intake and weight gain of dairy heifers fed sugar cane and grazing alfalfa. In: 46ª Reunião Anual da Sociedade Brasileira de Zootecnia, Maringá, PR, Brazil. 3p. 1–3. Sociedade Brasileira de Zootecnia, Brazil, 2009.
64. Santana, D.F.Y.; Lira, M.A.; Santos, M.V.F.; Ferreira, M.A.; Santos, D.C.; Mello, A.C.L.; Dubeux Júnior, J.C.B.; Araujo, G.G.L. Dry matter intake and performance of Girolando and Guzerá heifers and Guzerá under supplementation in caatinga, during the rainy season, in Pernambuco, Brazil. *Rev Bras Zootec* **2010**, 39, 2148–2154. https://doi.org/10.1590/S1516-35982010001000007
65. Santos, S.A.; Campos, J.M.S.; Valadares Filho, S.C.; Detmann, E.; Oliveira, A.S.; Souza, S.M. Productive performance of growing dairy heifers fed corn silage and soybean or cottonseed meal based concentrate. *Rev Bras Zootec* **2010**, 39, 638–647. https://doi.org/10.1590/S1516-35982010000300025
66. Siécola Júnior, S.; Bitencourt, L.L.; Melo, L.Q.; Silveira, V.A.; Lopes, N.M.; Silva, J.R.M.; Pereira, R.A.N.; Pereira, M.N. Deleafed sugarcane and performance of heifers and dairy cows. *Arq Bras Med Vet Zootec* **2014**, 66, 219–228. https://doi.org/10.1590/S0102-09352014000100030
67. Silva, F.F.; Aguiar, M.S.M.A.; Veloso, C.M.; Pires, A.J.V.; Bonomo, P.; Dutra, G.S.; Almeida, V.S.; Carvalho, G.G.P.; Silva, R.R.; Dias, A.M.; Ítavo, L.C.V. Performance of dairy heifers fed on elephantgrass silage added with different levels of cassava bagasse. *Arq Bras Med Vet Zootec* **2006**, 58, 205–211. https://doi.org/10.1590/S0102-09352006000200009
68. Silva, D.C. Metabolismo em novilhas Girolando com fenótipos divergentes para eficiência alimentar. Master's thesis, State University of Southwestern Bahia, Itapetinga, BA, Brazil, 2017.
69. Silva, A.L.; Detmann, E.; Dijkstra, J.; Pedroso, A.M.; Silva, L.H.P.; Machado, A.F.; Sousa, F.C.; Santos, G.B.; Marcondes, M.I. Effects of rumen-undegradable protein on intake, performance, and mammary gland development in prepubertal and pubertal dairy heifers. *J Dairy Sci* **2018**, 101, 5991–6001. http://doi.org/10.3168/jds.2017-13230
70. Sousa, M.G. Proteína degradável no rúmen em suplementos múltiplos para novilhas Girolandas à pasto. Master's thesis, Federal University of Jequitinhonha and Mucuri Valleys, Diamantina, MG, Brazil, 2018.
71. Souza, A.L.; Garcia, R.; Bernardino, F.S.; Campos, J.M.S.; Valadares Filho, S.C.; Cabral, L.S.; Gobbi, K.F. Coffee hulls in dairy heifers diet: intake, digestibility, and production. *Rev Bras Zootec* **2006**, 35, 921–927. https://doi.org/10.1590/S1516-35982006000300039
72. Souza, D.D. Farelo de mamona em dietas para novilhas leiteiras em pastejo. Doctor's thesis, State University of Southwestern Bahia, Itapetininga, BA, Brazil, 2018.
73. Teixeira, R.M.A.; Campos, J.M.S.; Valadares Filho, S.C.; Oliveira, A.S.; Assis, A.J.; Pina, D.S. Intake, digestibility and performance of dairy heifers fed coffee hulls replacing of corn silage. *Rev Bras Zootec* **2007**, 36, 968–977. https://doi.org/10.1590/S1516-35982007000400029
74. Teixeira, F.A.; Silva, F.F.; Bonomo, P.; Pires, A.J.V.; Nascimento, P.V.N.; Gonçalves Neto, J. Performance of dairy heifers grazing on *Urochloa decumbens* pastures deferred for two periods. *Acta Sci Anim Sci* **2014**, 36, 109–115. https://doi.org/10.4025/actascianimsci.v36i1.21759
75. Vieira, V.C.F. Associação do bagaço de cana-de-açúcar, palma forrageira e ureia com diferentes suplementos em dietas de novilhas da raça Holandesa. Master's thesis, Federal Rural University of Pernanbuco, Recife, PE, Brazil, 2006.
